# Arsenic Exposure and Neuropsychological Outcomes in Children: A Scoping Review

**DOI:** 10.3390/toxics13070542

**Published:** 2025-06-28

**Authors:** Leyre Notario-Barandiaran, Laura M. Compañ-Gabucio, Julia A. Bauer, Jesús Vioque, Margaret R. Karagas, Antonio J. Signes-Pastor

**Affiliations:** 1 Department of Epidemiology, Geisel School of Medicine, Dartmouth College, Hanover, NH 03756, USA; 2 Departamento de Patología y Cirugía, Universidad Miguel Hernández (UMH), 03550 Alicante, Spain; 3 Instituto de Investigación Sanitaria y Biomédica de Alicante (ISABIAL), 03010 Alicante, Spain; 4 Unidad de Epidemiología de la Nutrición, Departamento de Salud Pública, Historia de la Ciencia y Ginecología, Universidad Miguel Hernández (UMH), 03550 Alicante, Spain; 5 Centro de Investigación Biomédica en Red de Epidemiología y Salud Pública (CIBERESP), Instituto de Salud Carlos III, 28029 Madrid, Spain; 6 Division of Epidemiology and Biostatistics, School of Public Health, University of Illinois Chicago, Chicago, IL 60612-4394, USA

**Keywords:** arsenic, child development, child, review, speciation techniques, neurotoxicity

## Abstract

A child’s exposure to arsenic (As) can begin in utero through placental transfer to the fetus. There is a growing body of epidemiologic evidence suggesting an association between As exposure and neuropsychological development. Therefore, our objective was to describe the consequences of maternal and/or childhood As exposure on children’s neuropsychological development. We conducted a scoping review with a systematic search of the PubMed, Scopus, EMBASE, Web of Science, and PsycINFO databases. We included studies that assessed the association between maternal and/or childhood As exposure and neuropsychological development in children up to an average of 12 years of age. A total of 77 studies were included, most of which were published between 2020 and 2024 (44.1%), conducted in the United States of America (18.2%) and Bangladesh (16.9%), and involved participants with a median age of 6.6 years. Most studies performed cross-sectional analyses (51.9%) and assessed exposure to elements other than As (64.9%). Childhood was the most frequently studied exposure window (57.2%), and urine was the most commonly used biomarker of exposure (58.4%), followed by blood or serum (32.3%). Cognition was the most frequently evaluated neuropsychological domain (94.8%), followed by psychomotor function (40.3%) and social–emotional function (29.9%). Most studies reported evidence of a negative impact of As exposure on children’s neuropsychological development (73.7%), while some found no changes (27.3%) and a few suggested an improvement (1.3%). An important limitation is that most studies measured total urinary As without speciation into inorganic versus organic forms, which limits the validity of dose–response conclusions based on total arsenic concentrations. This review highlights the potential deleterious neuropsychological effects of maternal and/or childhood As exposure while also identifying areas where the evidence remains inconclusive.

## 1. Introduction

Arsenic (As) is a ubiquitous metalloid, and exposure to it poses a significant global health concern, impacting over 200 million people worldwide [1]. Humans are exposed to As through diverse sources, such as water, air, soil, and food [2]. In populations without occupational exposure to As, water and food are the main sources of exposure [3]. In the environment, As can be found in both organic and inorganic (iAs) forms, with iAs being the main toxic form of As [4]. The inorganic forms of As, including As^III^ and As^V^, are more readily absorbed by the gastrointestinal tract and have been associated with different health problems, such as cancer, cardiovascular disorders, and neurotoxicity, in the general population [5]. Exposure to As during pregnancy is particularly concerning, as this stage of life represents a uniquely vulnerable period in maternal health and fetal development [6]. Previous research shows that iAs can easily cross the placental barrier and reach fetal tissues, potentially leading to complications during pregnancy and adverse birth outcomes [7], such as spontaneous abortion, preterm birth, fetal death, and low birth weight [8]. While the precise mechanism through which iAs affects health remains unclear, some studies have suggested that iAs can interfere with physiological processes, including altering DNA and inhibiting DNA repair and cell proliferation processes [9]. Effects of prenatal As exposure on child neurological development stand out among the health concerns associated with exposure to As during pregnancy. Previous studies have shown the capacity of As to cross the blood–brain barrier [10], triggering several neurotoxic effects, such as neurotransmitter impairment, oxidative stress, brain cell apoptosis, and epigenetic modifications [11]. During late pregnancy and early childhood, the brain undergoes maximal plasticity and a series of complex events, including neurogenesis, myelination, and synaptic pruning [12,13]. Thus, minimizing iAs exposure during pregnancy is crucial to preserve optimal brain development in young children.

A previous investigation raised the possibility that As exposure during pregnancy adversely impacts child neurodevelopment in highly exposed populations [14]; however, in populations with relatively low exposure levels, studies are scarce, and results are inconsistent. For example, in a cross-sectional study carried out in Spain, a country with relatively low levels of iAs contamination, iAs exposure (average urinary concentration of 4.85 μg/L) at 4–5 years of age was inversely associated with children’s neuropsychological outcomes, specifically, fine and gross motor function [15]. Another study carried out in the Health Outcomes and Measures of the Environment (HOME) study found an inverse association between urinary iAs concentrations during pregnancy and children’s cognition at 3, 5, and 8 years of age [16]. However, the detrimental effect of iAs exposure on children’s neuropsychological development is not well-established, as some studies find no evidence of such an effect [17,18]. Several published reviews on As exposure during pregnancy and/or childhood have been conducted to examine preterm birth [19], the effects on the central nervous system among children exposed to lower As levels [20], the health impacts of drinking As-contaminated water in children [21], and the association between As exposure, adverse pregnancy outcomes, and infant mortality [22]. One review examined the scientific evidence on neurodevelopment and behavioral disorders in children exposed to As, cadmium, and manganese [23]. One systematic review summarizes the literature on factors that modify the associations between lead, methylmercury, manganese, and As and neurodevelopment in children [24], while another review examines epidemiological studies on the effect of chronic As exposure in drinking water on children’s intelligence quotient [25]. However, none of the previous reviews cover the broad scope of this scoping review, which considers various periods of As exposure and includes the literature from recent years, during which there has been a considerable increase in research. This rise in research may partly be attributed to the 2019 publication of the Healthy Babies Bright Futures (HBBF) report, which highlighted the widespread presence of toxic metals, including As, in baby foods and their potential impact on neurodevelopment, raising concerns about cognitive deficits and lower IQ in children [26,27].

Due to the disparity of results in studies on pre- and perinatal periods and infant As exposure and child neurodevelopment, as well as the existing knowledge gaps, we conducted a scoping review of epidemiological studies. The aim of this scoping review is to provide a structured overview of the existing scientific evidence addressing the following question: What are the observed neuropsychological consequences of As exposure during the prenatal and perinatal periods and early childhood?

## 2. Materials and Methods

We conducted a scoping review in accordance with the PRISMA Extension for Scoping Reviews (PRISMA-ScR) [28] (Table S1). We chose this approach because scoping reviews are best suited to addressing broad research questions [29,30]. Although this study is not a systematic review, we adhered to the standards outlined in the Cochrane Handbook Version 6.4, 2023 [31], to ensure content completeness, methodological transparency, and scientific rigor. We have not published a protocol of this review or registered it on PROSPERO or any other similar scientific website.

### 2.1. Search Strategy

Two of the authors conducted a literature search in five databases on 4 June 2024. We selected these databases based on the results of a previous study [32], which identified an optimal combination of databases for covering the broadest range of published evidence. This combination includes multidisciplinary databases, such as PubMed (MEDLINE), Scopus, EMBASE, and Web of Science, complemented by a specialized database relevant to our research question; in this case, PsycINFO.

The search strategy, developed under the supervision of a research and education librarian from the Biomedical Library at Dartmouth, utilized a combination of terms with the Boolean operators AND and OR, following the PICO structure. Specifically, we used a combination of search terms covering three main components: population (children, infants, prenatal or postnatal periods), exposure (arsenic and its inorganic forms), and outcomes (neuropsychological issues). The full search strategy applied across all five databases is detailed Table S2. The same search strategy was applied across all five databases consulted. We did not apply time restrictions or other filters in any of the databases consulted.

### 2.2. Eligibility Criteria

Articles had to meet the following inclusion criteria to be included in this scoping review. Language of publication: Spanish and/or English; study design: experimental and/or observational; study population: children aged, on average, ≤12 years without neurodevelopmental disorders (Autism Spectrum Disorder (ASD), Attention-Deficit/Hyperactivity Disorder (ADHD), intellectual disability, language disability, communication disorders, neurodevelopmental motor disorders, and specific learning disorders). This age range was selected because it represents a critical developmental phase characterized by rapid physical and psychological transitions between childhood and adolescence [33,34]. By focusing on children aged 12 or younger, we aim to address the whole of childhood [35]. Study exposure variable: exposure to As from diet, water, or industry; study outcomes: neuropsychological development. This concept is broad and has a variety of definitions. To make this scoping review more replicable, we used the Joan Forns et al. [36] definition of neuropsychological development. Based on this definition, neuropsychological development includes three different domains: psychomotor (fine and gross motor abilities), cognition (visuospatial abilities, language and/or communication, learning and memory, attention, and executive function), and socio-emotional (social competence, attachment, adaptive behavior, and emotional competence). Another inclusion criterion was that the studies had to have the full text available. No studies were excluded for this reason, as all articles were obtained through our university library services with their support. We applied and tested all inclusion criteria manually.

### 2.3. Study Selection and Screening

We carried out study selection and screening manually through Microsoft Excel version 16.0 (Microsoft Excel, Redmond, WA, USA). The titles found in the searches of each of the five databases were downloaded into Microsoft Excel (Redmond, WA, USA). We merged all of the titles into a single Excel sheet to eliminate duplicates. Once all duplicates were removed, we began the screening process. This process was conducted in three phases: title screening, abstract screening, and full text screening. Different Excel sheets were used for each screening phase. All three screening phases were carried out completely, carefully, and independently by two authors, discarding only those articles that we were certain did not meet the inclusion criteria. Discrepancies arising during the study selection process regarding the inclusion or exclusion of an article were resolved by a third author. The study selection flow was displayed graphically using the PRISMA diagram [37].

### 2.4. Data Extraction and Synthesis

Data were extracted in an Excel database, which was prepared before beginning the bibliographic search to guarantee transparency and avoid information manipulation. While this study adopts a scoping review approach, we adhered to certain guidelines outlined in the Cochrane Handbook [31] for the preparation and layout of tables to ensure comprehensive content coverage. We performed a narrative and graphical synthesis of the extracted information, using tables and figures whenever possible. We completed one table with key general information from the included articles. We elaborated different graphs and figures with R software version 4.0.4 (R Foundation for Statistical Computing, Vienna, Austria). to describe some of the variables in greater depth. On the one hand, we used Sankey plots, an increasingly utilized type of graph in the field of health sciences. These plots enable us to visualize changes over time and the evolution of specific variables in relation to a particular event [38]. This type of graph has been used in previous published review papers [39,40,41,42] to present the main characteristics of the articles in a visual and more comprehensible way. Three of the researchers were responsible for extracting data and performing the narrative and graphical synthesis of the information in the results section. The quality of the included studies was not assessed, as this is not required in scoping reviews [28].

## 3. Results

**Figure 1:**
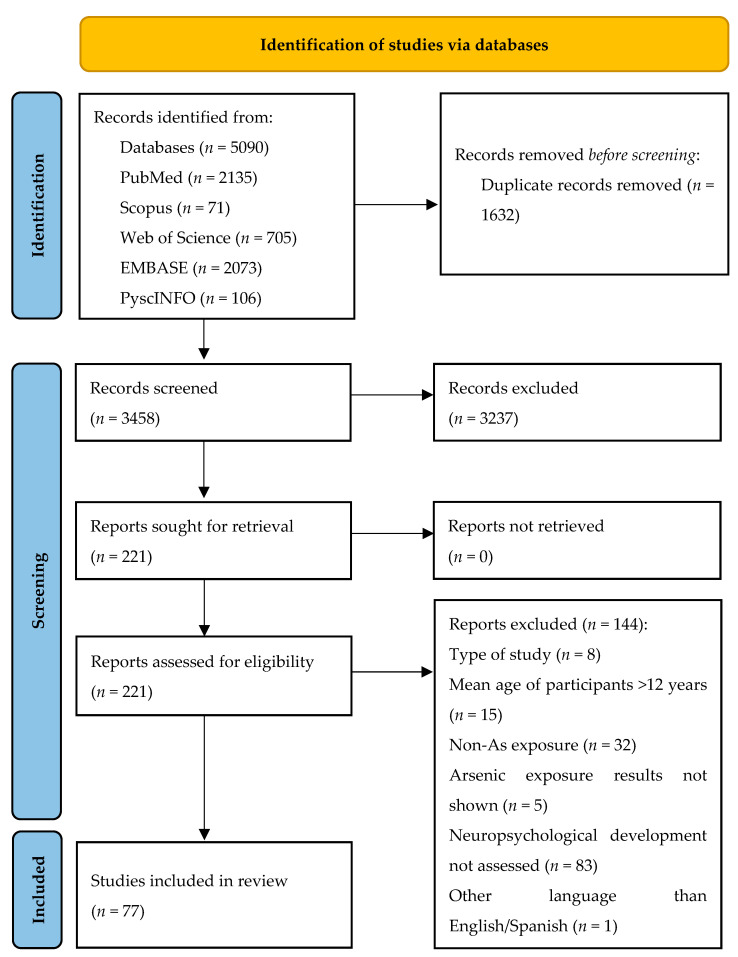
Flowchart of the study selection process.

The initial search across selected databases was enhanced by excluding duplicate records. Subsequently, the remaining records underwent screening, identifying 221 for eligibility assessment. Finally, 77 original studies were included to conduct the current scoping review on evaluating the evidence on As exposure during the pre- and perinatal periods or childhood and its impact on various aspects of neuropsychological development (Figure 1).

### 3.1. Main Characteristics of the Included Studies

**Figure 2:**
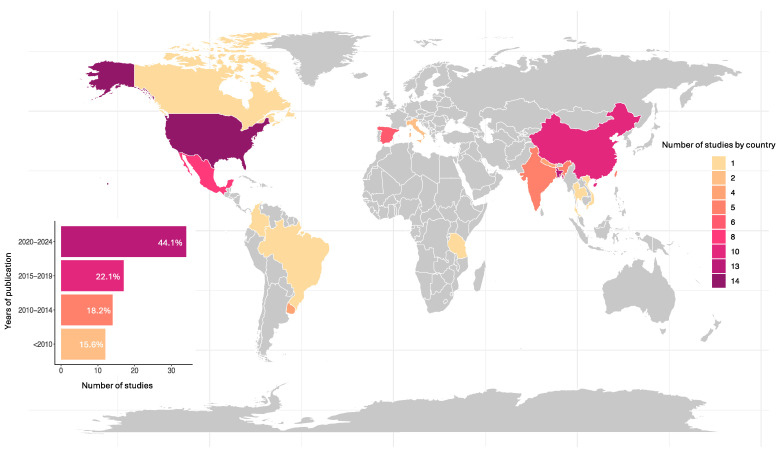
Geographic and temporal distribution of included studies (*n* = 77).

The included studies, spanning from 1981 to 2024, feature participants from 16 countries, with the United States of America (USA) [16,33,43,44,45,46,47,48,49,50,51,52,53,54], Bangladesh [14,17,18,55,56,57,58,59,60,61,62,63,64], China [65,66,67,68,69,70,71,72,73,74], and Mexico [75,76,77,78,79,80,81,82] being the most prevalent contributors (Figure 2).

Notably, 56 (72.7%) studies explicitly focused on As exposure, as indicated by the inclusion of the word “arsenic” or its abbreviation in their stated aims. Among the selected studies, 35 (45.4%) used participants from well-defined study populations and 19 different cohorts, including cVEDA [83], the Healthy Baby Cohort Study in China [70], MABC [67], the Mining and Health prospective longitudinal study [84], the PIPA project [85], PROGRESS [75,77,78], the Taiwan Birth Panel Study (TBPS) [86], the HOME study [16], HEALS [57,59,60,64], INMA [15,87,88,89], MINIMat [14,17,18,63], MIREC [90], NHBCS [46,49,52], the PRISM pregnancy cohort [48,53], the Sheyang Mini Birth Cohort Study (SMBCS) [71,74], the C8 Health Project [33], the Navajo Birth Cohort Study [76], Project Viva [54], and Environmental Pollution in Young Children [73].

**Figure 3:**
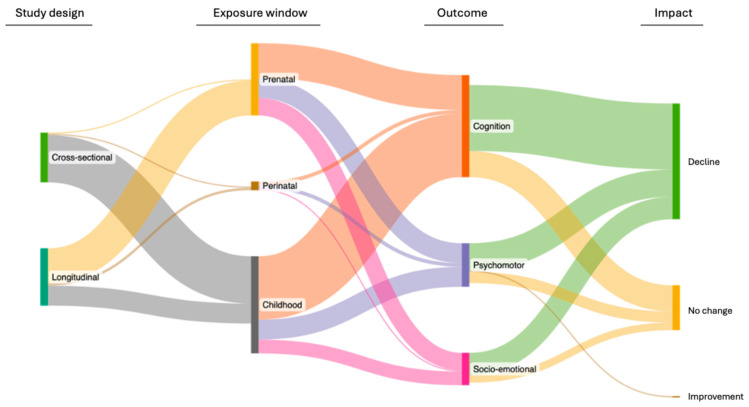
Sankey plot from the included studies in the scoping review (*n* = 77).

Among the study designs, 40 (51.9%) were cross-sectional, and 37 (48.1%) were longitudinal [14,16,17,18,46,47,48,49,52,53,54,55,56,57,63,67,68,69,70,71,74,75,76,77,78,84,85,86,87,88,89,90,91,92,93,94,95] (Table 1). Among longitudinal studies, 16 (43.2%) evaluated childhood exposure [14,16,17,49,52,54,56,57,63,68,71,74,75,88,91,94], 2 (5.4%) assessed perinatal exposure [86,94], and 28 (75.7%) examined prenatal exposure [14,17,18,46,47,48,49,52,53,55,63,67,68,69,70,74,75,76,77,78,84,85,87,89,90,92,93,95] to As. Among cross-sectional studies, 38 (95.0%) evaluated childhood exposure [15,23,33,43,44,45,50,51,58,59,60,61,62,64,65,66,73,79,80,81,82,83,96,97,98,99,100,101,102,103,104,105,106,107,108,109,110,111], 1 (2.5%) assessed perinatal exposure [112], and 2 (5.0%) examined prenatal exposure to As [72,110] (Figure 3). All studies included boys and girls, with a median final sample size of 301 participants.

### 3.2. Exposure Assessment and Analytical Techniques

Among the selected studies, 50 (64.9%) studies evaluated elements beyond As, with lead assessed in 42 (54.6%) of the selected studies, cadmium in 26 (33.8%), manganese in 24 (31.7%), mercury in 16 (20.8%), and selenium in 13 (16.8%). Other elements assessed include copper, barium, beryllium, zinc, iodine, chromium, antimony, fluorine, aluminum, cesium, and lithium, each in varying proportions. The most common analytical techniques were inductively coupled plasma mass spectrometry (ICP-MS) and atomic absorption spectrometry (AAS), reported in 55 (71.4%) and 18 (23.4%) studies, respectively. Among the identified studies, 15 (19.5%) reported the use of chromatography equipment to perform speciation analysis [14,15,16,17,52,62,70,78,90,96,97,98,102,107,108], and 3 (20.0%) of them did not provide data on As species concentration [14,17,78].

### 3.3. Markers of Exposure

Urine was the most commonly used biomarker of internal exposure, appearing in 45 (58.4%) studies [14,15,16,17,18,23,48,52,53,57,58,59,60,61,62,63,64,65,66,70,71,73,74,75,76,79,80,81,82,83,84,87,89,90,96,97,98,102,103,105,106,107,108,109,111]. Blood and serum followed, used in 25 (32.5%) of the included studies [47,54,55,57,59,60,63,64,65,67,68,69,72,76,77,78,85,86,91,92,93,95,101,106,111]. Other biological matrices included hair [33,44,45,50,51,63,94,99,100,101,104,106,112] and toenails [46,49,96] or fingernails [94,99,110,112], which were also frequently employed. Several studies additionally analyzed water samples as a proxy for iAs exposure. Less frequently used biomarkers included meconium [94] and the placenta [88]. Notably, 23 (29.9%) studies utilized multiple biomarkers to capture exposure more comprehensively [49,57,58,59,60,61,62,63,64,65,66,76,82,94,96,99,101,103,104,105,106,111,112] (Figures S1–S3).

Among the studies using urine as a biomarker, 36 (80.0%) evaluated childhood exposure [14,15,16,17,23,52,57,58,59,60,61,62,63,64,65,66,71,73,74,75,79,80,81,82,83,96,97,98,102,103,105,106,107,108,109,111], 15 (33.3%) evaluated prenatal exposure [14,17,18,48,52,53,63,70,74,75,76,84,87,89,90], and 6 (13.3%) addressed both periods [14,17,52,63,74,75]. Regarding blood/serum biomarker studies, 12 (48.0%) evaluated childhood exposure [54,57,59,60,63,64,65,68,91,101,106,111], 14 (56.0%) evaluated prenatal exposure [47,55,63,67,68,69,72,76,77,78,85,92,93,95], 1 (4.0%) evaluated perinatal exposure [86], and 2 (8.0%) evaluated both prenatal and childhood exposure [63,68]. Of the 16 studies using urinary biomarkers that provided data on As speciation, the most common approach was summing iAs metabolites, with iAs median concentrations ranging from 3.63 µg/L [16] to 55.2 µg/L [79]. In studies reporting total urinary As, average concentrations ranged from 8.3 µg/L [84,106] to 181.9 µg/L [58]. For blood As, average levels ranged from 1.33 µg/L [92] to 9.46 µg/L [85]. Hair As concentrations ranged from 0.19 mg/kg [94] to 2.72 mg/kg [98], while As levels in water ranged from 5.8 µg/L [82] to 857 µg/L [105].

Of the 77 studies reviewed, 14 (18.2%) did not specify a primary As source. Of the 63 that did, 20 (25.9%) examined drinking water, 21 (27.3%) focused on dietary sources, and 8 (10.4%) assessed both pathways. In addition, 14 (18.2%) studies identified mining and industrial emissions as the principal origins of As exposure.

### 3.4. Neuropsychological Function and Assessment Methods

In this scoping review, we used the conceptual framework developed by Forns et al. [36] to define the different neuropsychological functional domains (cognition, psychomotor, and socio-emotional). Cognition was a major functional domain of investigation (*n* = 73, 94.8%) (Figure 3 and Figure S1). Within this subset, 44 (60.3%) explored language and communication [14,15,16,17,33,43,44,47,51,53,54,55,57,61,62,63,64,67,68,70,71,76,80,81,82,84,85,86,87,88,91,92,94,95,96,97,98,100,101,103,104,109,110,112], 27 (37.0%) examined executive function [15,18,23,33,43,44,53,57,63,64,67,76,77,78,80,81,83,87,88,89,97,98,101,103,104,107,109], 26 (35.6%) investigated visuospatial abilities [14,15,33,43,44,45,51,54,57,61,62,63,64,68,71,80,81,82,87,88,97,98,101,103,107,109], 24 (32.9%) studies evaluated the subdomain of attention [23,33,44,46,49,53,60,69,72,73,74,79,80,81,88,89,93,97,98,101,104,106,109,111], and 22 (30.1%) focused on learning and memory [15,33,43,44,53,54,57,63,64,75,80,81,87,88,97,98,101,103,104,107,108,109].

The psychomotor functional domain was evaluated in 31 (40.2%) of the included studies (Figure 3 and Figure S2). Within this subset, 27 (87.1%) studies evaluated the subdomain of fine motor skills [15,17,18,44,45,47,52,54,56,59,67,70,76,84,85,86,87,88,91,92,94,95,96,97,101,103,112], 23 (74.2%) assessed gross motor abilities [15,17,18,47,59,67,69,70,72,76,84,85,86,87,88,91,92,93,94,95,96,97,112], and 1 (3.2%) examined general motor skills [110]. It is worth noting that 20 (64.5%) studies evaluated both fine and gross skills [15,17,18,47,59,67,70,76,84,85,86,87,88,91,92,94,95,96,97,112].

The socio-emotional domain was the least studied among the included articles, with only 23 (29.9%) focusing on this area (Figure 3 and Figure S3). Within this subset, 18 (78.26%) studies evaluated the subdomain social competence [33,44,46,49,60,67,73,74,76,84,85,86,90,106,110,111], 17 (73.9%) explored emotional competence [33,44,46,49,60,69,72,73,74,79,89,90,106,110,111], and 15 (65.2%) examined adaptive behavior [33,44,46,49,60,73,74,86,90,93,106,110,111]. No studies evaluated attachment.

Among the reviewed studies, a variety of tests were applied, demonstrating a diverse array of assessment methods. The Wechsler Intelligence Scale for Children (WISC) test was the most frequently used, appearing in 19 (24.7%) of the studies [14,16,33,43,44,57,61,62,63,64,68,71,80,81,82,90,103,106,109], followed by the Bayley Scales of Infant Development (BSID), which were used in 14 (18.2%) studies [16,17,18,55,56,70,91,92,94,95,96,100,110,112], Raven’s Progressive Matrices (RPM) in 6 (7.8%) studies [58,65,66,99,103,105], and the Behavior Assessment System for Children (BASC) in 5 (6.5%) studies [33,44,46,49,90]. Only six tests evaluated all three domains simultaneously: BSID, the Comprehensive Developmental Inventory for Infants and Toddlers (CDIIT), the Malawi Developmental Assessment Tool (MDAT), the Denver Developmental Screening Test II (DDST-II), Neonatal Behavioral Neurological Assessments (NBNA), and the Ages and Stages Questionnaire Inventory (ASQ). Most of these tests are recommended for children over 5 years old, with only a few focusing on early neuropsychological assessment (BSID, ASQ, NBNA, CDIIT, and DDST-II) (Table S3). Multiple tests were applied in 25 (32.5%) studies [16,17,18,23,33,44,49,51,53,54,58,75,79,80,83,90,101,102,103,104,106,107,108,109,111].

### 3.5. Arsenic Exposure and Neuropsychological Function

Among the included studies, 57 (74.0%) reported neuropsychological decline, 21 (27.3%) reported no neuropsychological change, and 1 (1.3%) reported neuropsychological improvement.

In the studies evaluating cognition (Figure S1), 52 (71.2%) reported a cognitive decline, 23 (31.5%) were cohort studies, and 29 (39.73%) were cross-sectional, with most focusing on exposure during childhood. Urine was the most commonly used biomarker of As exposure. These adverse effects were predominantly reported in studies conducted in countries like Bangladesh [14,55,56,57,58,61,62,63,64], Mexico [75,76,77,78,79,80,81,82], China [66,67,68,69,70,72,74], and the USA [16,33,43,44,49,51,53]. Among the 16 studies that included As speciation, 15 (93.7%) evaluated cognition. Of these, 11 (73.3%) reported significant inverse associations between iAs and cognitive performance [15,16,62,70,76,79,80,87,90,96,107], and 4 (26.7%) found no statistically significant relationship [97,98,102,108]. Cognitive decline was primarily observed in the domains of language and communication (n = 33, 45.2%) [14,15,16,33,43,44,51,53,55,57,61,62,63,64,67,68,70,76,80,81,82,84,85,87,88,94,96,97,101,103,104,110,112], executive function (n = 22, 30.1%) [15,23,33,43,44,53,57,63,64,67,76,78,80,81,83,87,88,97,101,103,104,107], visuospatial skills (n = 21, 28.77%) [14,15,33,43,44,51,57,61,62,63,64,68,80,81,82,87,88,97,101,103,107], attention (n = 18, 24.66%), and learning and memory (n = 18, 24.7%) [15,33,43,44,53,57,63,64,75,80,81,87,88,97,101,103,104,107]. The remaining studies assessing cognition did not report statistically significant associations with As exposure.

In the studies evaluating psychomotor skills (Figure S2), 22 (70.9%) reported a decline, 9 (29.0%) observed no change, and 1 (3.2%) reported an improvement [97]. Among the 22 studies reporting a decline, 7 (31.8%) performed As speciation, confirming that the negative associations were driven specifically by iAs [15,52,70,76,87,96,97]. Of these 22 decline studies, 12 (54.6%) were cohort studies and 10 (45.5%) were cross-sectional in design and primarily focused on childhood exposure, although several also assessed prenatal and perinatal exposure. Urine and blood were the most frequently used biomarkers of As exposure. Among these 22 (70.9%) studies, 18 (81.8%) assessed fine motor abilities [15,44,52,56,59,67,70,76,84,85,87,88,94,96,97,101,103,112] and 16 (72.7%) assessed gross motor abilities [15,59,67,69,70,72,76,84,85,87,88,93,94,96,97,112]. Notably, the single study reporting a beneficial effect examined the association between the organic As species arsenobetaine and fine motor function [97].

Regarding socio-emotional development (Figure S3), 18 (78.3%) studies reported a negative impact, with 6 (26.1%) reporting no changes. Of the 18 studies reporting socio-emotional decline, 10 (55.6%) were cohorts and 8 (44.4%) cross-sectional and focused mainly on prenatal exposure, with urine and blood used as the primary biomarkers of exposure. Of the 18 studies documenting a socio-emotional decline, 3 (16.7%) included As speciation analyses and confirmed that the adverse effects were specifically linked to iAs [76,79,90]. In the studies that reported socio-emotional decline, 14 (77.8%) focused on social competence [33,44,48,49,50,67,74,76,84,85,90,106,110,111], 11 (61.1%) on adaptive behavior [33,44,48,49,50,74,90,93,106,110,111], and 13 (72.2%) on emotional competence [33,44,48,49,50,69,72,74,79,90,106,110,111].

### 3.6. Confounding Factors

To account for socioeconomic status, various variables, such as parental education level and family income, were incorporated into 66 (85.7%) studies. Gestational age, maternal age at delivery, or child age at assessment were considered potentially confounding factors in 62 (80.5%) studies. Forty-six studies included child sex as a potential confounding factor. Maternal pre-pregnancy BMI was regarded as a potentially confounding factor in 23 (29.9%) studies. Maternal IQ was considered in 14 (18.2%) studies. Parity was considered in 14 (18.2%) studies. Home Observation for Measurement of the Environment (HOME) was accounted for in 11 (14.3%) studies. Smoking status, including passive smoking, parental smoking, and serum cotinine levels, was identified as a potentially confounding factor in 13 (16.9%) studies [16,46,49,52,65,68,70,71,74,88,98,106]. Children’s head circumference was included in 9 (11.7%) studies. Additionally, diet variables, such as calorie-adjusted fish and rice intake and a healthy eating index, were recognized as potentially confounding factors in 10 (13.0%) studies [15,23,46,49,54,74,86,99,101]. We identified two studies that did not adjust for any potentially confounding factors [66,104].

## 4. Discussion

In this scoping review, we summarize the current state of environmental epidemiologic studies regarding As exposure and neuropsychological impact in children. Most of the evidence, 74% of the included studies, supports a negative association between As exposure and children’s neuropsychological development. Except for one study suggesting a positive link [97], the rest found no association [17,18,45,46,47,54,60,65,71,73,74,86,89,91,92,95,98,100,102,108,109]. The majority of the studies included in this review were published between 2019 and 2024, the most recent four years, suggesting growing scientific interest in this topic.

The USA, followed by Bangladesh, were the countries where most of the studies included in this review were conducted. For several years, there has been concern regarding exposure to As through diet and water in the USA [113,114]. The USA was included among the countries most severely affected by As contamination in groundwater, being considered one of the high-income countries with the highest As contamination levels in water [115]. However, the concentrations are typically lower than those found in Asian countries, such as Bangladesh, where contamination in drinking water is considered a massive public health issue [116], as aquifers are severely affected and millions of people consume water with high levels of As [117]. Concentrations of As measured in drinking water varied dramatically across the included studies, from 5.8 µg/L in a Mexican cohort [82] to over 857 µg/L in Bihar, India [104], more than 85 times above the World Health Organization (WHO) recommendation of 10 µg/L. In Mexico, water As at 5.8 µg/L was nonetheless linked to lower scores in performance, verbal, and full IQ scores, suggesting that harms may occur even below the 10 µg/L threshold [82]. In Bangladesh and India, where drinking water arsenic frequently exceeded 50 µg/L and in many cases surpassed 100 µg/L, multiple studies reported dose-related declines in verbal IQ, processing speed, and memory scores [58,63,99,104]. All of this evidence may explain why the USA and Bangladesh were the most studied countries in this review and why water was one of the most frequently reported sources of As exposure in the included studies.

Dietary sources of As, particularly rice-based infant cereals and toddler foods, emerged as a second critical exposure pathway. The 2019 HBBF Alliance report first quantified that baby foods with iAs levels of 100–150 µg/kg contributed substantially to an estimated loss of over 11 million IQ points across USA children aged 0–24 months, with rice products alone accounting for roughly 20 percent of total IQ reductions [26]. These concentrations exceed the USA Food and Drug Administration’s action level of 100 µg/kg, yet our review found that neurodevelopmental effects were apparent at mean dietary As levels as low as 120 µg/kg. Conversely, studies focusing on arsenobetaine, a non-toxic organic As species prevalent in fish and seafood, reported no adverse cognitive outcomes and even suggested modest cognitive benefits attributable to omega-3 fatty acids [15,97,98].

Urinary total As served as the most widely used biomarker, reflecting combined exposure from water and diet [118,119]. A recent review highlights As in urine as an effective biomarker of dietary and water consumption exposure capable of detecting both inorganic and organic compounds. Consequently, the review concludes that the number of epidemiological studies utilizing urinary analysis to assess As exposure is currently escalating and poised for further expansion in the future [120]. Remarkably, in a USA birth cohort with mean urinary iAs of just 3.6 µg/L, researchers observed significant reductions in processing speed and working memory [16]. In populations with urinary As exceeding the reference value of <50 µg/L, which is considered the upper limit of acceptable exposure in non-occupationally exposed populations, the associations with decreased IQ, attention deficits, and impaired school readiness were both consistent and robust [58,79]. It should be noted that the <50 µg/L urinary As value is a commonly used reference level but not an official regulatory limit for non-occupational exposure. In contrast, cohorts with urinary arsenic under 10 µg/L generally showed no significant neuropsychological impairments, hinting at a possible threshold [60,109]; however, inter-study variability in cognitive assessments and confounding adjustment underscores that this potential cutoff requires confirmation.

Another key point discussed in the aforementioned review is the importance of As speciation in urine. As speciation is crucial due to the significant differences in toxicity among As species. Generally, inorganic As species are highly toxic, whereas organic As species are excreted unchanged in urine and therefore considered non-toxic [121]. Consequently, using urinary As for speciation analysis is regarded as a reference method for determining the specific As species to which individuals are exposed, enabling targeted interventions to reduce exposure and mitigate health effects. Nevertheless, it should be noted that few of the included studies conducted As speciation, possibly because these methods are relatively new and specific to identifying only certain arsenicals, which may not be applicable to other As species [122]. The effect of As exposure during pregnancy and/or childhood on children’s neuropsychological function was negatively associated (74%) in the majority of the included studies. These results are in line with those presented in a review carried out by Ortiz-Garcia et al., whose main objective was to determine the impact of As exposure on maternal and fetal health [8]. In the previous review, maternal As exposure during pregnancy was identified as a critical window of concern, as As can easily cross the placental barrier and lead to fetal alterations, such as low birth weight and congenital anomalies [8]. Similarly, a systematic review of 92 studies conducted in Latin America highlighted the harmful effects of As exposure during pregnancy and/or childhood on several health issues, such as kidney injury, short gestational age, pulmonary illness, and decreased cognitive function in children [123]. In our review, most of the included studies analyzed the effects of maternal and/or childhood As exposure on children’s cognitive function, which is frequently assessed in neuropsychological evaluations [36]. One possible reason most studies analyzed in this review focus on cognitive function is the role of As as a neurotoxic agent. As has been shown to cross the blood–brain barrier, potentially causing direct effects on the central nervous system [124,125,126]. Exposure to As during pregnancy may affect the central nervous system by altering neuronal plasticity, disrupting neural networks, and causing oxidative stress [125]. Nonetheless, our review also identified studies with evidence of null and/or protective effects of As exposure during pregnancy and/or infancy on neuropsychological function in children. These findings suggest that the evidence may still be inconclusive and that further research is needed. Importantly, however, the positive association found in one of the papers included in the review was derived from arsenobetaine exposure, a non-toxic organic form of As that is widely used in epidemiological studies as an indicator of fish/seafood consumption in children [15,119,127]. Fish and seafood are also foods that stand out for being rich in essential nutrients crucial for brain development, including proteins, vitamins, and highlighting omega-3 long-chain polyunsaturated fatty acids [128]. High concentrations of docosahexaenoic acid (DHA) and eicosapentaenoic acid (EPA) are found in the brain, and these are important components of neuronal cell membranes; they support synaptic plasticity and have anti-inflammatory properties [129,130]. As speciation can help tease apart the effects of beneficial nutrients in fish and seafood for neurodevelopment, whereas assessing total As could lead to misclassification of exposure, especially in populations with high fish consumption.

The present review has certain limitations. We cannot rule out selection bias, which is inherent in all studies. Our inclusion criteria, such as limiting articles to only two languages, may have increased this bias. However, one of these languages was English, which is the most commonly used language in scientific research. Additionally, using only five databases may have led to overlooking relevant articles. We should note that these databases were selected based on the recommendations of Bramer et al. [32] for an optimal combination of databases, which helps ensure comprehensive coverage of the available scientific literature. We encountered challenges in establishing our search strategy due to the broad nature of neuropsychological development and the lack of a standard definition, which complicated the selection of search terms. As a result, we may have overlooked some relevant terms during the search process, potentially introducing selection bias. To mitigate this limitation, we used the definition proposed by Joan Forns et al. [36] for neuropsychological development in epidemiological studies to guide our selection of search terms, thus enhancing the replicability of this review.

Another inherent limitation in review papers is publication bias, which can indirectly contribute to selection bias. This occurs when scientific journals do not publish studies with null results, leading to their exclusion from review articles. In our study, we categorized results as neuropsychological improvement, decline, or no change to clearly convey outcomes. However, it is important to recognize that “no change” results do not imply the absence of a neuropsychological impact; rather, they indicate that the observed effect was not statistically significant based on our study’s criteria. This necessary categorization highlights primary findings but may overlook meaningful, non-significant trends. Taken together, publication bias and the limitations inherent in our reporting criteria underscore the need for caution when interpreting non-significant findings, as future research with different methodologies or larger sample sizes may provide additional insights. An additional critical limitation concerns As exposure assessment. Most included studies report only total urinary As without speciation into inorganic versus organic forms, which restricts the validity of dose–response interpretations. Finally, we acknowledge that the quality of the included articles was not assessed. Although assessing article quality is not a mandatory requirement for scoping reviews [131], the lack of such an assessment could have led us to include low-quality articles. To mitigate this limitation, we have provided figures and narrative descriptions that include information on indicators closely related to methodological quality, such as conflicts of interest, funding sources, and limitations reported by the authors of the included articles. This information is intended to help readers interpret the results presented in this scoping review.

We want to further highlight certain strengths of this scoping review. It provides a synthesis of information based on a large number of studies (*n* = 77) on a specific and relevant topic, namely, As exposure and its effect on neuropsychological function in childhood. In this context, it is important to note that children are considered a vulnerable study population, as disruptions during this critical stage can compromise development throughout life, increasing the scientific interest of this work. Another strength of this study is our use of a structured, detailed, and systematic methodology, ensuring the replicability of our review. Additionally, we used a combination of databases to search for articles, which is deemed optimal according to the scientific literature [32]. Finally, the greatest strength of this work and of scoping reviews in general is their ability to identify knowledge gaps. This identification helps other researchers target future investigations and complement the existing evidence. The primary knowledge gaps identified in this scoping review are the following: 1. the limited number of studies conducted in low-income countries, excluding Bangladesh, such as African and Latin American countries, and 2. the insufficient number of articles performing As speciation analysis given the critical importance of understanding exposure to different As species. Without proper speciation analysis, there is a risk of misinterpreting results and potentially underestimating or overestimating health risks, which could lead to ineffective or misguided public health interventions. Thirdly, the lack of comparability among studies due to the wide array of tests used to assess neuropsychological function makes it nearly impossible to conduct meta-analyses to evaluate the overall effects across studies.

## 5. Conclusions

This scoping review indicates that As exposure during prenatal, perinatal, and early-childhood stages is frequently linked to neurodevelopmental decline, particularly in cognitive function, although a minority of studies report null findings. The widespread use of urine as a biomarker of As exposure, coupled with heterogeneous speciation methods, sampling times, and neuropsychological instruments, has limited comparability and prevented clear dose–response characterization. Future work that harmonizes exposure assessment with iAs speciation, employs standardized developmental batteries across cognitive, motor, and socio-emotional domains, and follows cohorts longitudinally from gestation to school age will be essential to establish safe exposure thresholds, inform regulatory standards, and guide targeted public health interventions.

## Figures and Tables

**Table 1:** Main characteristics and findings of the included studies (*n* = 77).

Study Design	*n* (%)
Cross-sectional	40 (51.9)
Longitudinal	37 (48.1)
Number of participants	median (IQR)
	301 (148–439)
Age of participants in months	median (IQR)
Minimum age	60.0 (30.6–72.0)
Median age	78.8 (44.6–81.0)
Maximum age	96 (45.3–131.4)
Evaluated metal exposure	*n* (%)
As	27 (35.1)
As + other metals	50 (64.9)
Exposure window	*n* (%)
Perinatal/prenatal	23 (29.9)
Childhood	44 (57.2)
Both	10 (12.9)
Main source of exposure	*n* (%)
Water	20 (25.9)
Food	21 (27.3)
Food and water	8 (10.4)
Industry	14 (18.2)
Not described	14 (18.2)
Neuropsychological assessment	*n* (%)
Single test	52 (67.5)
Multiple tests	25 (32.5)
Main exposure effects	*n* (%)
Beneficial	1 (1.3)
Harmful	57 (74.0)
Null	21 (27.3)

As, arsenic; IQR, interquartile range; *n*, number.

## Data Availability

No new data were created or analyzed in this study. The data used in this study are included in the article/Supplementary Material.

## Supplementary Materials

The following supporting information can be downloaded at https://www.mdpi.com/article/10.3390/toxics13070542/s1, Table S1: Preferred Reporting Items for Systematic Reviews and Meta-Analyses Extension for Scoping Reviews (PRISMA-ScR) Checklist; Table S2: Search strategy and databases consulted; Table S3: Characteristics of the most used assessment test for neuropsychological function; Figure S1: Sankey plot from the included studies assessing the cognition functional domain; Figure S2: Sankey plot from the included studies assessing the psychomotor functional domain; Figure S3: Sankey plot from the included studies assessing the socio-emotional functional domain.

## Abbreviations

The following abbreviations are used in this manuscript:

As	Arsenic
DHA	Docosahexaenoic acid
EPA	Eicosapentaenoic acid
iAs	Inorganic arsenic
WHO	World Health Organization

## References

[1] Fatoki J.O., Badmus J.A. (2022). Arsenic as an Environmental and Human Health Antagonist: A Review of Its Toxicity and Disease Initiation. J. Hazard. Mater. Adv..

[2] Arslan B., Djamgoz M.B.A., Akün E. (2017). ARSENIC: A Review on Exposure Pathways, Accumulation, Mobility and Transmission into the Human Food Chain. Rev. Environ. Contam. Toxicol..

[3] Chen Q.Y., Costa M. (2021). Arsenic: A Global Environmental Challenge. Annu. Rev. Pharmacol. Toxicol..

[4] Arcella D., Cascio C., Gómez Ruiz J. (2021). Ángel Chronic Dietary Exposure to Inorganic Arsenic. EFSA J..

[5] Ganie S.Y., Javaid D., Hajam Y.A., Reshi M.S. (2023). Arsenic Toxicity: Sources, Pathophysiology and Mechanism. Toxicol. Res..

[6] Miodovnik A. (2011). Environmental Neurotoxicants and Developing Brain. Mt. Sinai J. Med. A J. Transl. Pers. Med..

[7] Punshon T., Davis M.A., Marsit C.J., Theiler S.K., Baker E.R., Jackson B.P., Conway D.C., Karagas M.R. (2015). Placental Arsenic Concentrations in Relation to Both Maternal and Infant Biomarkers of Exposure in a US Cohort. J. Expo. Sci. Environ. Epidemiol..

[8] Ortiz-Garcia N.Y., Ramírez A.I.C., Juarez K., Galindo J.B., Briceño G., Martinez E.C. (2023). Maternal Exposure to Arsenic and Its Impact on Maternal and Fetal Health: A Review. Cureus.

[9] Thomas D.J. (2021). Arsenic Methylation-Lessons from Three Decades of Research. Toxicology.

[10] Jin Y., Xi S., Li X., Lu C., Li G., Xu Y., Qu C., Niu Y., Sun G. (2006). Arsenic Speciation Transported Through the Placenta from Mother Mice to Their Newborn Pups. Environ. Res..

[11] Chakraborty A., Ghosh S., Biswas B., Pramanik S., Nriagu J., Bhowmick S. (2022). Epigenetic Modifications from Arsenic Exposure: A Comprehensive Review. Sci. Total Environ..

[12] Likhar A., Patil M.S. (2022). Importance of Maternal Nutrition in the First 1000 Days of Life and Its Effects on Child Development: A Narrative Review. Cureus.

[13] Scher M.S. (2021). “The First Thousand Days” Define a Fetal/Neonatal Neurology Program. Front. Pediatr..

[14] Hamadani J.D., Tofail F., Nermell B., Gardner R., Shiraji S., Bottai M., Arifeen S.E., Huda S.N., Vahter M. (2011). Critical Windows of Exposure for Arsenic-Associated Impairment of Cognitive Function in Pre-School Girls and Boys: A Population-Based Cohort Study. Int. J. Epidemiol..

[15] Signes-Pastor A.J., Vioque J., Navarrete-Muñoz E.M., Carey M., Garc-Villarino M., Fernández-Somoano A., Tardón A., Santa-Marina L., Irizar A., Casas M. (2019). Inorganic Arsenic Exposure and Neuropsychological Development of Children of 4–5 Years of Age Living in Spain. Environ. Res..

[16] Signes-Pastor A.J., Romano M.E., Jackson B., Braun J.M., Yolton K., Chen A., Lanphear B., Karagas M.R. (2022). Associations of Maternal Urinary Arsenic Concentrations During Pregnancy with Childhood Cognitive Abilities: The HOME Study. Int. J. Hyg. Environ. Health.

[17] Hamadani J.D., Grantham-McGregor S.M., Tofail F., Nermell B., Fängström B., Huda S.N., Yesmin S., Rahman M., Vera-Hernández M., Arifeen S.E. (2010). Pre- and Postnatal Arsenic Exposure and Child Development at 18 Months of Age: A Cohort Study in Rural Bangladesh. Int. J. Epidemiol..

[18] Tofail F., Vahter M., Hamadani J.D., Nermell B., Huda S.N., Yunus M., Rahman M., Grantham-McGregor S.M. (2009). Effect of Arsenic Exposure During Pregnancy on Infant Development at 7 Months in Rural Matlab, Bangladesh. Environ. Health Perspect..

[19] Margiana R., Alshahrani S.H., Kayumova D., Alawadi A.H.R., Hjazi A., Alsalamy A., Qasim Q.A., Juyal A., Garousi N. (2023). Association Between Maternal Exposure to Arsenic by Drinking Water During Pregnancy and Risk of Preterm Birth: A Systematic Review and Meta-Analysis. Int. J. Environ. Health Res..

[20] Tsuji J.S., Garry M.R., Perez V., Chang E.T. (2015). Low-Level Arsenic Exposure and Developmental Neurotoxicity in Children: A Systematic Review and Risk Assessment. Toxicology.

[21] Majumdar K.K., Mazumder D.N.G. (2012). Effect of Drinking Arsenic-Contaminated Water in Children. Indian J. Public Health.

[22] Quansah R., Armah F.A., Essumang D.K., Luginaah I., Clarke E., Marfoh K., Cobbina S.J., Nketiah-Amponsah E., Namujju P.B., Obiri S. (2015). Association of Arsenic with Adverse Pregnancy Outcomes/Infant Mortality: A Systematic Review and Meta-Analysis. Environ. Health Perspect..

[23] Rodríguez-Barranco M., Gil F., Hernández A.F., Alguacil J., Lorca A., Mendoza R., Gómez I., Molina-Villalba I., González-Alzaga B., Aguilar-Garduño C. (2016). Postnatal Arsenic Exposure and Attention Impairment in School Children. Cortex.

[24] Bauer J.A., Fruh V., Howe C.G., White R.F., Henn B.C. (2020). Associations of Metals and Neurodevelopment: A Review of Recent Evidence on Susceptibility Factors. Curr. Epidemiol. Rep..

[25] Hasanvand M., Mohammadi R., Khoshnamvand N., Jafari A., Palangi H.S., Mokhayeri Y. (2020). Dose-Response Meta-Analysis of Arsenic Exposure in Drinking Water and Intelligence Quotient. J. Environ. Health Sci. Eng..

[26] Jane H., Charlotte B. (2019). What’s in My Baby’s Food?.

[27] Ryan K., Meghan L. (2019). Results of Lifetime IQ Decrement Analysis from Dietary Exposures to Lead and Inorganic Arsenic for Children 0 to <2 Years of Age.

[28] Tricco A.C., Lillie E., Zarin W., O’Brien K.K., Colquhoun H., Levac D., Moher D., Peters M.D.J., Horsley T., Weeks L. (2018). PRISMA Extension for Scoping Reviews (PRISMA-ScR): Checklist and Explanation. Ann. Intern. Med..

[29] Gough D., Thomas J., Oliver S. (2012). Clarifying Differences Between Review Designs and Methods. Syst. Rev..

[30] Munn Z., Peters M.D.J., Stern C., Tufanaru C., McArthur A., Aromataris E. (2018). Systematic Review or Scoping Review? Guidance for Authors When Choosing Between a Systematic or Scoping Review Approach. BMC Med. Res. Methodol..

[31] Higgins J.P. (2023). Cochrane Handbook for Systematic Reviews of Interventions Version 6.4 (Updated August 2023).

[32] Bramer W.M., Rethlefsen M.L., Kleijnen J., Franco O.H. (2017). Optimal Database Combinations for Literature Searches in Systematic Reviews: A Prospective Exploratory Study. Syst. Rev..

[33] Stein C.R., Wu H., Bellinger D.C., Smith D.R., Wolff M.S., Savitz D.A. (2022). Exposure to Metal Mixtures and Neuropsychological Functioning in Middle Childhood. Neurotoxicology.

[34] Mah V.K., Ford-Jones E.L. (2012). Spotlight on Middle Childhood: Rejuvenating the ‘Forgotten Years’. Paediatr. Child Health.

[35] Voss M.L., Claeson M., Bremberg S., Peterson S.S., Alfvén T., Ndeezi G. (2023). The Missing Middle of Childhood. Glob. Health Action.

[36] Forns J., Aranbarri A., Grellier J., Julvez J., Vrijheid M., Sunyer J. (2012). A Conceptual Framework in the Study of Neuropsychological Development in Epidemiological Studies. Neuroepidemiology.

[37] Page M.J., McKenzie J.E., Bossuyt P.M., Boutron I., Hoffmann T.C., Mulrow C.D., Shamseer L., Tetzlaff J.M., Akl E.A., Brennan S.E. (2021). The PRISMA 2020 Statement: An Updated Guideline for Reporting Systematic Reviews. BMJ.

[38] Otto E., Culakova E., Meng S., Zhang Z., Xu H., Mohile S., Flannery M.A. (2022). Overview of Sankey Flow Diagrams: Focusing on Symptom Trajectories in Older Adults with Advanced Cancer. J. Geriatr. Oncol..

[39] Gutiérrez-González E., García-Esquinas E., de Larrea-Baz N.F., Salcedo-Bellido I., Navas-Acien A., Lope V., Gómez-Ariza J.L., Pastor R., Pollán M., Pérez-Gómez B. (2019). Toenails as Biomarker of Exposure to Essential Trace Metals: A Review. Environ. Res..

[40] Ritschl V., Ferreira R.J.O., Santos E.J.F., Fernandes R., Juutila E., Mosor E., Santos-Costa P., Fligelstone K., Schraven L., Stummvoll G. (2021). Suitability for e-Health of Non-Pharmacological Interventions in Connective Tissue Diseases: Scoping Review with a Descriptive Analysis. RMD Open.

[41] Wibble T., Pansell T. (2023). Clinical Characteristics of Visual Motion Hypersensitivity: A Systematic Review. Exp. Brain Res..

[42] Jiang H., Wang W., Mei Y., Zhao Z., Lin B., Zhang Z. (2023). A Scoping Review of the Self-Reported Compassion Measurement Tools. BMC Public Health.

[43] Wasserman G.A., Liu X., Loiacono N.J., Kline J., Factor-Litvak P., van Geen A., Mey J.L., Levy D., Abramson R., Schwartz A. (2014). A Cross-Sectional Study of Well Water Arsenic and Child IQ in Maine Schoolchildren. Environ. Health.

[44] Wright R.O., Amarasiriwardena C., Woolf A.D., Jim R., Bellinger D.C. (2006). Neuropsychological Correlates of Hair Arsenic, Manganese, and Cadmium Levels in School-Age Children Residing Near a Hazardous Waste Site. Neurotoxicology.

[45] Marlowe M., Stellern J., Errera J., Moon C. (1985). Main and Interaction Effects of Metal Pollutants on Visual-Motor Performance. Arch. Environ. Health.

[46] Bauer J.A., Romano M.E., Jackson B.P., Bellinger D., Korrick S., Karagas M.R. (2024). Associations of Perinatal Metal and Metalloid Exposures with Early Child Behavioral Development over Time in the New Hampshire Birth Cohort Study. Expo. Health.

[47] Cottrell J., Nelson C., Waldron C., Bergeron M., Samson A., Valentovic M. (2023). Effect of Umbilical Cord Essential and Toxic Elements, Thyroid Levels, and Vitamin d on Childhood Development. Biomed. Pharmacother..

[48] Cowell W., Colicino E., Levin-Schwartz Y., Enlow M.B., Amarasiriwardena C., Andra S.S., Gennings C., Wright R.O., Wright R.J. (2021). Prenatal Metal Mixtures and Sex-Specific Infant Negative Affectivity. Environ. Epidemiol..

[49] Doherty B.T., Romano M.E., Gui J., Punshon T., Jackson B.P., Karagas M.R., Korrick S.A. (2020). Periconceptional and Prenatal Exposure to Metal Mixtures in Relation to Behavioral Development at 3 Years of Age. Environ. Epidemiol..

[50] Marlowe M., Cossairt A., Moon C., Errera J., MacNeel A., Peak R., Ray J., Schroeder C. (1985). Main and Interaction Effects of Metallic Toxins on Classroom Behavior. J. Abnorm. Child. Psychol..

[51] Moon C., Marlowe M., Stellern J., Errera J. (1985). Main and Interaction Effects of Metallic Pollutants on Cognitive Functioning. J. Learn. Disabil..

[52] Butler E.E., Karagas M.R., Demidenko E., Bellinger D.C., Korrick S.A. (2023). In Utero Arsenic Exposure and Early Childhood Motor Development in the New Hampshire Birth Cohort Study. Front. Epidemiol..

[53] Rosa M.J., Pedretti N.F., Goldson B., Mathews N., Merced-Nieves F., Xhani N., Enlow M.B., Gershon R., Ho E., Huddleston K. (2024). Integrating Data Across Multiple Sites in the Northeastern United States to Examine Associations Between a Prenatal Metal Mixture and Child Cognition. Am. J. Epidemiol..

[54] Thilakaratne R., Lin P.I.D., Rifas-Shiman S.L., Landero J., Wright R.O., Bellinger D., Oken E., Cardenas A. (2024). Cross-Sectional and Prospective Associations of Early Childhood Circulating Metals with Early and Mid-Childhood Cognition in the Project Viva Cohort. Environ. Res..

[55] Valeri L., Mazumdar M.M., Bobb J.F., Henn B.C., Rodrigues E., Sharif O.I.A., Kile M.L., Quamruzzaman Q., Afroz S., Golam M. (2017). The Joint Effect of Prenatal Exposure to Metal Mixtures on Neurodevelopmental Outcomes at 20–40 Months of Age: Evidence from Rural Bangladesh. Environ. Health Perspect..

[56] Rodrigues E.G., Bellinger D.C., Valeri L., Hasan M.O.S.I., Quamruzzaman Q., Golam M., Kile M.L., Christiani D.C., Wright R.O., Mazumdar M. (2016). Neurodevelopmental Outcomes Among 2- to 3-Year-Old Children in Bangladesh with Elevated Blood Lead and Exposure to Arsenic and Manganese in Drinking Water. Environ. Health A Glob. Access Sci. Source.

[57] Wasserman G.A., Liu X., Parvez F., Factor-Litvak P., Kline J., Siddique A.B., Shahriar H., Uddin M.N., van Geen A., Mey J.L. (2016). Child Intelligence and Reductions in Water Arsenic and Manganese: A Two-Year Follow-up Study in Bangladesh. Environ. Health Perspect.

[58] Nahar M.N., Inaoka T., Fujimura M. (2014). A Consecutive Study on Arsenic Exposure and Intelligence Quotient (IQ) of Children in Bangladesh. Environ. Health Prev. Med..

[59] Parvez F., Wasserman G.A., Factor-Litvak P., Liu X., Slavkovich V., Siddique A.B., Sultana R., Sultana R., Islam T., Levy D. (2011). Arsenic Exposure and Motor Function Among Children in Bangladesh. Environ. Health Perspect..

[60] Khan K., Factor-Litvak P., Wasserman G.A., Liu X., Ahmed E., Parvez F., Slavkovich V., Levy D., Mey J., van Geen A. (2011). Manganese Exposure from Drinking Water and Children’s Classroom Behavior in Bangladesh. Environ. Health Perspect..

[61] Wasserman G.A., Liu X., Parvez F., Ahsan H., Factor-Litvak P., Kline J., van Geen A., Slavkovich V., Loiacono N.J., Levy D. (2007). Water Arsenic Exposure and Intellectual Function in 6-Year-Old Children in Araihazar, Bangladesh. Environ. Health Perspect..

[62] Wasserman G.A., Liu X., Parvez F., Ahsan H., Factor-Litvak P., van Geen A., Slavkovich V., LoIacono N.J., Cheng Z., Hussain I. (2004). Water Arsenic Exposure and Children’s Intellectual Function in Araihazar, Bangladesh. Environ. Health Perspect..

[63] Vahter M., Skröder H., Rahman S.M., Levi M., Hamadani J.D., Kippler M. (2020). Prenatal and Childhood Arsenic Exposure Through Drinking Water and Food and Cognitive Abilities at 10 Years of Age: A Prospective Cohort Study. Environ. Int..

[64] Wasserman G.A., Liu X., Parvez F., Factor-Litvak P., Ahsan H., Levy D., Kline J., van Geen A., Mey J., Slavkovich V. (2011). Arsenic and Manganese Exposure and Children’s Intellectual Function. Neurotoxicology.

[65] Pan S., Lin L., Zeng F., Zhang J., Dong G., Yang B., Jing Y., Chen S., Zhang G., Yu Z. (2018). Effects of Lead, Cadmium, Arsenic, and Mercury Co-Exposure on Children’s Intelligence Quotient in an Industrialized Area of Southern China. Environ. Pollut..

[66] Wang S.-X., Wang Z.-H., Cheng X.-T., Li J., Sang Z.-P., Zhang X.-D., Han L.-L., Qiao X.-Y., Wu Z.-M., Wang Z.-Q. (2007). Arsenic and Fluoride Exposure in Drinking Water: Children’s IQ and Growth in Shanyin County, Shanxi Province, China. Environ. Health Perspect..

[67] Liang C., Wu X., Huang K., Yan S., Li Z., Xia X., Pan W., Sheng J., Tao R., Tao Y. (2020). Domain- and Sex-Specific Effects of Prenatal Exposure to Low Levels of Arsenic on Children’s Development at 6 Months of Age: Findings from the Ma’anshan Birth Cohort Study in China. Environ. Int..

[68] Wang Y., Wang Y., Yan C. (2022). Gender Differences in Trace Element Exposures with Cognitive Abilities of School-Aged Children: A Cohort Study in Wujiang City, China. Environ. Sci. Pollut. Res. Int..

[69] Yu X.-D., Yan C.-H., Shen X.-M., Tian Y., Cao L.-L., Yu X.-G., Zhao L., Liu J.-X. (2011). Prenatal Exposure to Multiple Toxic Heavy Metals and Neonatal Neurobehavioral Development in Shanghai, China. Neurotoxicol Teratol..

[70] Chen H., Zhang H., Wang X., Wu Y., Zhang Y., Chen S., Zhang W., Sun X., Zheng T., Xia W. (2023). Prenatal Arsenic Exposure, Arsenic Metabolism and Neurocognitive Development of 2-Year-Old Children in Low-Arsenic Areas. Environ. Int..

[71] Zhou T., Guo J., Zhang J., Xiao H., Qi X., Wu C., Chang X., Zhang Y., Liu Q., Zhou Z. (2020). Sex-Specific Differences in Cognitive Abilities Associated with Childhood Cadmium and Manganese Exposures in School-Age Children: A Prospective Cohort Study. Biol. Trace Elem. Res..

[72] Wang B., Liu J., Liu B., Liu X., Yu X. (2018). Prenatal Exposure to Arsenic and Neurobehavioral Development of Newborns in China. Environ. Int..

[73] Ma J., Geng S., Sun Q., Zhang X., Han L., Yao X., Zhang B., Zhu L., Wen J. (2023). Exposure to Metal Mixtures and Young Children’s Growth and Development: A Biomonitoring-Based Study in Eastern China. Ecotoxicol. Environ. Saf..

[74] Dai Y., Lu H., Zhang J., Ding J., Wang Z., Zhang B., Qi X., Chang X., Wu C., Zhou Z. (2023). Sex-Specific Associations of Maternal and Childhood Urinary Arsenic Levels with Emotional Problems Among 6-Year-Age Children: Evidence from a Longitudinal Cohort Study in China. Ecotoxicol. Environ. Saf..

[75] Merced-Nieves F.M., Chelonis J., Pantic I., Schnass L., Téllez-Rojo M.M., Braun J.M., Paule M.G., Wright R.J., Wright R.O., Curtin P. (2022). Prenatal Trace Elements Mixture Is Associated with Learning Deficits on a Behavioral Acquisition Task Among Young Children. New Dir. Child. Adolesc. Dev..

[76] Nozadi S.S., Li L., Luo L., Mackenzie D., Erdei E., Du R., Roman C.W., Hoover J., O’donald E., Burnette C. (2021). Prenatal Metal Exposures and Infants’ Developmental Outcomes in a Navajo Population. Int. J. Environ. Res. Public Health.

[77] de Water E., Curtin P., Gennings C., Chelonis J.J., Paule M., Bixby M., McRae N., Svensson K., Schnaas L., Pantic I. (2022). Prenatal Metal Mixture Concentrations and Reward Motivation in Children. Neurotoxicology.

[78] Levin-Schwartz Y., Gennings C., Schnaas L., Chávez M.D.C.H., Bellinger D.C., Téllez-Rojo M.M., Baccarelli A.A., Wright R.O. (2019). Time-Varying Associations Between Prenatal Metal Mixtures and Rapid Visual Processing in Children. Environ. Health.

[79] Roy A., Kordas K., Lopez P., Rosado J.L., Cebrian M.E., Vargas G.G., Ronquillo D., Stoltzfus R.J. (2011). Association Between Arsenic Exposure and Behavior Among First-Graders from Torreón, Mexico. Environ. Res..

[80] Rosado J.L., Ronquillo D., Kordas K., Rojas O., Alatorre J., Lopez P., Garcia-Vargas G., Caamaño M.C., Cebrián M.E., Stoltzfus R.J. (2007). Arsenic Exposure and Cognitive Performance in Mexican Schoolchildren. Environ. Health Perspect..

[81] Calderón J., Navarro M.E., Jimenez-Capdeville M.E., Santos-Diaz M.A., Golden A., Rodriguez-Leyva I., Borja-Aburto V., Díaz-Barriga F. (2001). Exposure to Arsenic and Lead and Neuropsychological Development in Mexican Children. Environ. Res..

[82] Rocha-Amador D., Navarro M.E., Carrizales L., Morales R., Calderón J. (2007). Decreased Intelligence in Children and Exposure to Fluoride and Arsenic in Drinking Water. Cad. Saude Publica.

[83] Vaidya N., Holla B., Heron J., Sharma E., Zhang Y., Fernandes G., Iyengar U., Spiers A., Yadav A., Das S. (2023). Neurocognitive Analysis of Low-Level Arsenic Exposure and Executive Function Mediated by Brain Anomalies Among Children, Adolescents, and Young Adults in India. JAMA Netw. Open.

[84] Nyanza E.C., Bernier F.P., Martin J.W., Manyama M., Hatfield J., Dewey D. (2021). Effects of Prenatal Exposure and Co-Exposure to Metallic or Metalloid Elements on Early Infant Neurodevelopmental Outcomes in Areas with Small-Scale Gold Mining Activities in Northern Tanzania. Environ. Int..

[85] Araujo M.S.D.A., de Figueiredo N.D., Froes-Asmus C.I.R. (2022). Prenatal Exposure to Metals and Neurodevelopment in Infants at Six Months: Rio Birth Cohort Study of Environmental Exposure and Childhood Development (PIPA Project). ISEE Conf. Abstr..

[86] Lin C.-C., Chen Y.-C., Su F.-C., Lin C.-M., Liao H.-F., Hwang Y.-H., Hsieh W.-S., Jeng S.-F., Su Y.-N., Chen P.-C. (2013). In Utero Exposure to Environmental Lead and Manganese and Neurodevelopment at 2 Years of Age. Environ. Res..

[87] Soler-Blasco R., Murcia M., Lozano M., Sarzo B., Esplugues A., Riutort-Mayol G., Vioque J., Lertxundi N., Marina L.S., Lertxundi A. (2022). Prenatal Arsenic Exposure, Arsenic Methylation Efficiency, and Neuropsychological Development Among Preschool Children in a Spanish Birth Cohort. Environ. Res..

[88] Freire C., Amaya E., Gil F., Fernández M.F., Murcia M., Llop S., Andiarena A., Aurrekoetxea J., Bustamante M., Guxens M. (2018). Prenatal Co-Exposure to Neurotoxic Metals and Neurodevelopment in Preschool Children: The Environment and Childhood (INMA) Project. Sci. Total Environ..

[89] Forns J., Fort M., Casas M., Cáceres A., Guxens M., Gascon M., Garcia-Esteban R., Julvez J., Grimalt J.O., Sunyer J. (2014). Exposure to Metals During Pregnancy and Neuropsychological Development at the Age of 4 Years. Neurotoxicology.

[90] Patti M.A., Kelsey K.T., MacFarlane A.J., Papandonatos G.D., Arbuckle T.E., Ashley-Martin J., Fisher M., Fraser W.D., Lanphear B.P., Muckle G. (2022). Maternal Folate Status and the Relation Between Gestational Arsenic Exposure and Child Health Outcomes. Int. J. Environ. Res. Public Health.

[91] Parajuli R.P., Fujiwara T., Umezaki M., Watanabe C. (2015). Home Environment and Cord Blood Levels of Lead, Arsenic, and Zinc on Neurodevelopment of 24 Months Children Living in Chitwan Valley, Nepal. J. Trace Elem. Med. Biol..

[92] Parajuli R.P., Fujiwara T., Umezaki M., Furusawa H., Watanabe C. (2014). Home Environment and Prenatal Exposure to Lead, Arsenic and Zinc on the Neurodevelopment of Six-Month-Old Infants Living in Chitwan Valley, Nepal. Neurotoxicol. Teratol..

[93] Parajuli R.P., Fujiwara T., Umezaki M., Watanabe C. (2013). Association of Cord Blood Levels of Lead, Arsenic, and Zinc with Neurodevelopmental Indicators in Newborns: A Birth Cohort Study in Chitwan Valley, Nepal. Environ. Res..

[94] Jiang C.B., Kao C.S., Chien L.C., Chen Y.J., Liao K.W. (2022). Associations Among Prenatal and Postnatal Arsenic, Lead, and Cadmium Exposures and Motor Development in 3-Year-Old Children: A Longitudinal Birth Cohort Study in Taiwan. Environ. Sci. Pollut. Res. Int..

[95] Parajuli R.P., Umezaki M., Fujiwara T., Watanabe C. (2015). Association of Cord Blood Levels of Lead, Arsenic, and Zinc and Home Environment with Children Neurodevelopment at 36 Months Living in Chitwan Valley, Nepal. PLoS ONE.

[96] Jiang C.B., Hsueh Y.M., Kuo G.L., Hsu C.H., Chang J.H., Chien L.C. (2018). Preliminary Study of Urinary Arsenic Concentration and Arsenic Methylation Capacity Effects on Neurodevelopment in Very Low Birth Weight Preterm Children Under 24 Months of Corrected Age. Medicine.

[97] Notario-Barandiaran L., Díaz-Coto S., Jimenez-Redondo N., Guxens M., Vrijheid M., Andiarena A., Irizar A., Riaño-Galan I., Fernández-Somoano A., Llop S. (2023). Latent Childhood Exposure to Mixtures of Metals and Neurodevelopmental Outcomes in 4–5-Year-Old Children Living in Spain. Expo. Health.

[98] Desai G., Barg G., Queirolo E.I., Vahter M., Peregalli F., Mañay N., Kordas K. (2018). A Cross-Sectional Study of General Cognitive Abilities Among Uruguayan School Children with Low-Level Arsenic Exposure, Potential Effect Modification by Methylation Capacity and Dietary Folate. Environ. Res..

[99] Manju R., Hegde A.M., Parlees P., Keshan A. (2017). Environmental Arsenic Contamination and Its Effect on Intelligence Quotient of School Children in a Historic Gold Mining Area Hutti, North Karnataka, India: A Pilot Study. J. Neurosci. Rural Pract..

[100] Kordas K., Ardoino G., Coffman D.L., Queirolo E.I., Ciccariello D., Mañay N., Ettinger A.S. (2015). Patterns of Exposure to Multiple Metals and Associations with Neurodevelopment of Preschool Children from Montevideo, Uruguay. J. Environ. Public Health.

[101] la Ossa C.A.D., Ramírez-Giraldo A.F., Arroyo-Alvis K., Marrugo-Negrete J., Díez S. (2023). Neuropsychological Effects and Cognitive Deficits Associated with Exposure to Mercury and Arsenic in Children and Adolescents of the Mojana Region, Colombia. Environ. Res..

[102] Kunwittaya S., Ruksee N., Khamnong T., Jiawiwatkul A., Kleebpung N., Chumchua V., Plitponkarnpim A., Nopparat C., Permpoonputtana K. (2022). Inorganic Arsenic Contamination and the Health of Children Living Near an Inactive Mining Site: Northern Thailand. EXCLI J..

[103] von Ehrenstein O.S., Poddar S., Yuan Y., Mazumder D.G., Eskenazi B., Basu A., Hira-Smith M., Ghosh N., Lahiri S., Haque R. (2007). Children’s Intellectual Function in Relation to Arsenic Exposure. Epidemiology.

[104] Kumar A., Rahman M.S., Kumar R., Ali M., Niraj P.K., Srivastava A., Singh S.K., Ghosh A.K. (2019). Arsenic Contamination in Groundwater Causing Impaired Memory and Intelligence in School Children of Simri Village of Buxar District of Bihar. J. Ment. Health Hum. Behav..

[105] Ghosh S.B., Chakraborty D., Mondal N.K. (2017). Effect of Arsenic and Manganese Exposure on Intellectual Function of Children in Arsenic Stress Area of Purbasthali, Burdwan, West Bengal. Expo. Health.

[106] Renzetti S., Cagna G., Calza S., Conversano M., Fedrighi C., Forte G., Giorgino A., Guazzetti S., Majorani C., Oppini M. (2021). The Effects of the Exposure to Neurotoxic Elements on Italian Schoolchildren Behavior. Sci. Rep..

[107] Desai G., Barg G., Vahter M., Queirolo E.I., Peregalli F., Mañay N., Millen A.E., Yu J., Kordas K. (2020). Executive Functions in School Children from Montevideo, Uruguay and Their Associations with Concurrent Low-Level Arsenic Exposure. Environ. Int..

[108] Desai G., Barg G., Vahter M., Queirolo E.I., Peregalli F., Mañay N., Millen A.E., Yu J., Browne R.W., Kordas K. (2020). Low Level Arsenic Exposure, b-Vitamins, and Achievement Among Uruguayan School Children. Int. J. Hyg. Environ. Health.

[109] Lucchini R.G., Guazzetti S., Renzetti S., Conversano M., Cagna G., Fedrighi C., Giorgino A., Peli M., Placidi D., Zoni S. (2019). Neurocognitive Impact of Metal Exposure and Social Stressors Among Schoolchildren in Taranto, Italy. Environ. Health A Glob. Access Sci. Source.

[110] Egwunye J., Cardoso B.R., Braat S., Ha T., Hanieh S., Hare D., Duan A.X., Doronila A., Tran T., Tuan T. (2022). The Role of Fingernail Selenium in the Association Between Arsenic, Lead and Mercury and Child Development in Rural Vietnam: A Cross-Sectional Analysis. Br. J. Nutr..

[111] Huang C., Pan S., Chin W., Hsu J., Guo Y.L. (2023). Urinary Heavy Metals and Attention-Deficit/Hyperactivity Symptoms of Preschool Children: A Mixed-Exposure Analysis. Ecotoxicol. Environ. Saf..

[112] Kao C.S., Fan Y.T., Chien L.C., Liao K.W., Chang J.H., Hsu C.H., Chen Y.J., Jiang C.B. (2023). Effects of Preterm Birth and Postnatal Exposure to Metal Mixtures on Neurodevelopment in Children at 24 Months of Age. Environ. Sci. Pollut. Res. Int..

[113] Wilson D. (2015). Arsenic Consumption in the United States. J. Environ. Health.

[114] Nachman K.E., Ginsberg G.L., Miller M.D., Murray C.J., Nigra A.E., Pendergrast C.B. (2017). Mitigating Dietary Arsenic Exposure: Current Status in the United States and Recommendations for an Improved Path Forward. Sci. Total Environ..

[115] Shaji E., Santosh M., Sarath K.V., Prakash P., Deepchand V., Divya B.V. (2021). Arsenic Contamination of Groundwater: A Global Synopsis with Focus on the Indian Peninsula. Geosci. Front..

[116] Rahman M.A., Rahman A., Khan M.Z.K., Renzaho A.M.N. (2018). Human Health Risks and Socio-Economic Perspectives of Arsenic Exposure in Bangladesh: A Scoping Review. Ecotoxicol. Environ. Saf..

[117] Rahaman M.S., Mise N., Ichihara S. (2022). Arsenic Contamination in Food Chain in Bangladesh: A Review on Health Hazards, Socioeconomic Impacts and Implications. Hyg. Environ. Health Adv..

[118] Wongsasuluk P., Chotpantarat S., Siriwong W., Robson M. (2021). Human Biomarkers Associated with Low Concentrations of Arsenic (as) and Lead (Pb) in Groundwater in Agricultural Areas of Thailand. Scientific Reports.

[119] Martinez-Morata I., Sobel M., Tellez-Plaza M., Navas-Acien A., Howe C.G., Sanchez T.R. (2023). A State-of-the-Science Review on Metal Biomarkers. Curr. Environ. Health Rep..

[120] Yoshinaga J. (2022). Urinary Arsenic as a Biomarker: Speciation Analysis for the Assessment of Dietary Exposure. Biomarkers in Disease: Methods, Discoveries and Applications.

[121] Jomova K., Jenisova Z., Feszterova M., Baros S., Liska J., Hudecova D., Rhodes C.J., Valko M. (2011). Arsenic: Toxicity, Oxidative Stress and Human Disease. J. Appl. Toxicol..

[122] Virk R.K., Garla R., Kaushal N., Bansal M.P., Garg M.L., Mohanty B.P. (2023). The Relevance of Arsenic Speciation Analysis in Health & Medicine. Chemosphere.

[123] Khan K.M., Chakraborty R., Bundschuh J., Bhattacharya P., Parvez F. (2020). Health Effects of Arsenic Exposure in Latin America: An Overview of the Past Eight Years of Research. Sci. Total Environ..

[124] Concha G., Vogler G., Lezcano D., Nermell B., Vahter M. (1998). Exposure to Inorganic Arsenic Metabolites During Early Human Development. Toxicol. Sci. Off. J. Soc. Toxicol..

[125] Tolins M., Ruchirawat M., Landrigan P. (2014). The Developmental Neurotoxicity of Arsenic: Cognitive and Behavioral Consequences of Early Life Exposure. Ann. Glob. Health.

[126] Cervantes G.I.V., Esquivel D.F.G., Ortega D.R., Ayala T.B., Chávez L.A.R., López-López H.E., Salazar A., Flores I., Pineda B., Gómez-Manzo S. (2023). Mechanisms Associated with Cognitive and Behavioral Impairment Induced by Arsenic Exposure. Cells.

[127] Jones M.R., Tellez-Plaza M., Vaidya D., Grau M., Francesconi K.A., Goessler W., Guallar E., Post W.S., Kaufman J.D., Navas-Acien A. (2016). Estimation of Inorganic Arsenic Exposure in Populations with Frequent Seafood Intake: Evidence from MESA and NHANES. Am. J. Epidemiol..

[128] Nevins J.E.H., Donovan S.M., Snetselaar L., Dewey K.G., Novotny R., Stang J., Taveras E.M., Kleinman R.E., Bailey R.L., Raghavan R. (2021). Omega-3 Fatty Acid Dietary Supplements Consumed During Pregnancy and Lactation and Child Neurodevelopment: A Systematic Review. J. Nutr..

[129] Sherzai D., Moness R., Sherzai S., Sherzai A. (2022). A Systematic Review of Omega-3 Fatty Acid Consumption and Cognitive Outcomes in Neurodevelopment. Am. J. Lifestyle Med..

[130] Basak S., Mallick R., Duttaroy A.K. (2020). Maternal Docosahexaenoic Acid Status During Pregnancy and Its Impact on Infant Neurodevelopment. Nutrients.

[131] Peters M.D.J., Marnie C., Tricco A.C., Pollock D., Munn Z., Alexander L., McInerney P., Godfrey C.M., Khalil H. (2020). Updated Methodological Guidance for the Conduct of Scoping Reviews. JBI Evid. Synth..

