# Supplementary material for: Arsenic Exposure and Neuropsychological Outcomes in Children: A Scoping Review
Source: Toxics. 2025 Jun 28;13(7):542. doi: 10.3390/toxics13070542 (PMC12300646; doi:10.3390/toxics13070542)
Supplement: Supplementary file 1 [file toxics-13-00542-s001.zip › toxics-3721006-supplementary.pdf]

<u>**Supplementary material**</u>

Arsenic exposure and neuropsychological outcomes in children: A scoping review

Table S1: Preferred Reporting Items for Systematic Reviews and Meta-Analyses Extension for Scoping Reviews (PRISMA-ScR) Checklist.

SECTION	ITEM	PRISMA-ScR CHECKLIST ITEM	REPORTED ON PAGE #
**TITLE**			
Title	1	Identify the report as a scoping review.	#Title page (0)
**ABSTRACT**			
Structured summary	2	Provide a structured summary that includes (as applicable): background, objectives, eligibility criteria, sources of evidence, charting methods, results, and conclusions that relate to the review questions and objectives.	#Abstract
**INTRODUCTION**			
Rationale	3	Describe the rationale for the review in the context of what is already known. Explain why the review questions/objectives lend themselves to a scoping review approach.	#2-3
Objectives	4	Provide an explicit statement of the questions and objectives being addressed with reference to their key elements (e.g., population or participants, concepts, and context) or other relevant key elements used to conceptualize the review questions and/or objectives.	#3
**METHODS**			
Protocol and registration	5	Indicate whether a review protocol exists; state if and where it can be accessed (e.g., a Web address); and if available, provide registration information, including the registration number.	#3
Eligibility criteria	6	Specify characteristics of the sources of evidence used as eligibility criteria (e.g., years considered, language, and publication status), and provide a rationale.	#3-4
Information sources*	7	Describe all information sources in the search (e.g., databases with dates of coverage and contact with authors to identify additional sources), as well as the date the most recent search was executed.	#3-4
Search	8	Present the full electronic search strategy for at least 1 database, including any limits used, such that it could be repeated.	#3, Table S2
Selection of sources of evidence†	9	State the process for selecting sources of evidence (i.e., screening and eligibility) included in the scoping review.	#4
Data charting process‡	10	Describe the methods of charting data from the included sources of evidence (e.g., calibrated forms or forms that have been tested by the team before their use, and whether data charting was done independently or in duplicate) and any processes for obtaining and confirming data from investigators.	#4
Data items	11	List and define all variables for which data were sought and any assumptions and simplifications made.	#4
Critical appraisal of individual sources of evidence§	12	If done, provide a rationale for conducting a critical appraisal of included sources of evidence; describe the methods used and how this information was used in any data synthesis (if appropriate).	#4,14
Synthesis of results	13	Describe the methods of handling and summarizing the data that were charted.	#4

SECTION	ITEM	PRISMA-ScR CHECKLIST ITEM	REPORTED ON PAGE #
**RESULTS**			
Selection of sources of evidence	14	Give numbers of sources of evidence screened, assessed for eligibility, and included in the review, with reasons for exclusions at each stage, ideally using a flow diagram.	#5, Figure 1
Characteristics of sources of evidence	15	For each source of evidence, present characteristics for which data were charted and provide the citations.	#5-11
Critical appraisal within sources of evidence	16	If done, present data on critical appraisal of included sources of evidence (see item 12).	#10-11
Results of individual sources of evidence	17	For each included source of evidence, present the relevant data that were charted that relate to the review questions and objectives.	#6-11, Figure S1, Figure S2, Figure S3
Synthesis of results	18	Summarize and/or present the charting results as they relate to the review questions and objectives.	#6-10, Table 1, Figure 2, Figure 3, Table S3, Figure S1, Figure S2, Figure S3
**DISCUSSION**			
Summary of evidence	19	Summarize the main results (including an overview of concepts, themes, and types of evidence available), link to the review questions and objectives, and consider the relevance to key groups.	#11-14
Limitations	20	Discuss the limitations of the scoping review process.	#13-14
Conclusions	21	Provide a general interpretation of the results with respect to the review questions and objectives, as well as potential implications and/or next steps.	#14
**FUNDING**			
Funding	22	Describe sources of funding for the included sources of evidence, as well as sources of funding for the scoping review. Describe the role of the funders of the scoping review.	#15

JBI = Joanna Briggs Institute; PRISMA-ScR = Preferred Reporting Items for Systematic Reviews and Meta-Analyses Extension for Scoping Reviews.

* Where *sources of evidence* (see second footnote) are compiled from, such as bibliographic databases, social media platforms, and Web sites.

† A more inclusive/heterogeneous term used to account for the different types of evidence or data sources (e.g., quantitative and/or qualitative research, expert opinion, and policy documents) that may be eligible in a scoping review as opposed to only studies. This is not to be confused with *information sources* (see first footnote).

‡ The frameworks by Arksey and O'Malley (6) and Levac and colleagues (7) and the JBI guidance (4, 5) refer to the process of data extraction in a scoping review as data charting.

§ The process of systematically examining research evidence to assess its validity, results, and relevance before using it to inform a decision. This term is used for items 12 and 19 instead of "risk of bias" (which is more applicable to systematic reviews of interventions) to include and acknowledge the various sources of evidence that may be used in a scoping review (e.g., quantitative and/or qualitative research, expert opinion, and policy document).

From: Tricco AC, Lillie E, Zarin W, O'Brien KK, Colquhoun H, Levac D, et al. PRISMA Extension for Scoping Reviews (PRISMAScR): Checklist and Explanation. Ann Intern Med. 2018;169:467–473. doi: 10.7326/M18-0850.

Table S2: Search strategy and databases consulted.

Databases	Search strategy 04-06-2024	Results
PubMed		
1	"arsenates"[MeSH Terms] OR "arsenates"[All Fields] OR "arsenic acid"[Supplementary Concept] OR "arsenic acid"[All Fields] OR "arsenate"[All Fields] OR "arsenic"[MeSH Terms] OR "arsenic"[All Fields] OR "arsenics"[All Fields] OR "arsenic s"[All Fields] OR "arsenicals"[MeSH Terms] OR "arsenicals"[All Fields] OR "arsenical"[All Fields] OR "arsenism"[All Fields] OR "arsenous"[All Fields] OR "arsenite"[Supplementary Concept] OR "arsenite"[All Fields] OR "arsenites"[MeSH Terms] OR "arsenites"[All Fields] OR "arsenates"[MeSH Terms] OR "arsenates"[All Fields] OR "arsenic acid"[Supplementary Concept] OR "arsenic acid"[All Fields] OR "arsenate"[All Fields] OR "arsenic"[MeSH Terms] OR "arsenic"[All Fields] OR "arsenics"[All Fields] OR "arsenic s"[All Fields] OR "arsenicals"[MeSH Terms] OR "arsenicals"[All Fields] OR "arsenical"[All Fields] OR "arsenism"[All Fields] OR "arsenous"[All Fields]	51,792
2	(("neurodevelopment*"[All Fields] OR "developmental disorder"[All Fields] OR "cognit*"[All Fields] OR ("neurocognition"[All Fields] OR "neurocognitive"[All Fields] OR "neurocognitively"[All Fields]) OR "intell*"[All Fields] OR ("academe"[All Fields] OR "academia"[MeSH Terms] OR "academia"[All Fields] OR "academic"[All Fields] OR "academics"[All Fields] OR "academic s"[All Fields] OR "academical"[All Fields] OR "academically"[All Fields]) OR "attention deficit hyperactivity disorder"[All Fields] OR ("attention deficit disorder with hyperactivity"[MeSH Terms] OR ("attention"[All Fields] AND "deficit"[All Fields] AND "disorder"[All Fields] AND "hyperactivity"[All Fields]) OR "attention deficit disorder with hyperactivity"[All Fields] OR "adhd"[All Fields]) OR ("autism s"[All Fields] OR "autisms"[All Fields] OR "autistic disorder"[MeSH Terms] OR ("autistic"[All Fields] AND "disorder"[All Fields]) OR "autistic disorder"[All Fields] OR "autism"[All Fields]) OR ("autistic disorder"[MeSH Terms] OR ("autistic"[All Fields] AND "disorder"[All Fields]) OR "autistic disorder"[All Fields] OR	7,334,273

	"autistic"[All Fields] OR "autistics"[All Fields] OR "autists"[All Fields]) OR ("autism s"[All Fields] OR "autisms"[All Fields] OR "autistic disorder"[MeSH Terms] OR ("autistic"[All Fields] AND "disorder"[All Fields]) OR "autistic disorder"[All Fields] OR "autism"[All Fields])) AND ("spectrum"[All Fields] OR "spectrum s"[All Fields] OR "spectrums"[All Fields]) AND ("disease"[MeSH Terms] OR "disease"[All Fields] OR "disorder"[All Fields] OR "disorders"[All Fields] OR "disorder s"[All Fields] OR "disordes"[All Fields])) OR ("asperger"[All Fields] OR "asperger s"[All Fields] OR "aspergers"[All Fields]) OR ("behavior"[MeSH Terms] OR "behavior"[All Fields] OR "behavioral"[All Fields] OR "behavioural"[All Fields] OR "behavior s"[All Fields] OR "behaviorally"[All Fields] OR "behaviour"[All Fields] OR "behaviourally"[All Fields] OR "behaviours"[All Fields] OR "behaviors"[All Fields] OR "pattern"[All Fields] OR "pattern s"[All Fields] OR "patternability"[All Fields] OR "patternable"[All Fields] OR "patterned"[All Fields] OR "patterning"[All Fields] OR "patternings"[All Fields] OR "patterns"[All Fields]) OR ("behavior"[MeSH Terms] OR "behavior"[All Fields] OR "behavioral"[All Fields] OR "behavioural"[All Fields] OR "behavior s"[All Fields] OR "behaviorally"[All Fields] OR "behaviour"[All Fields] OR "behaviourally"[All Fields] OR "behaviours"[All Fields] OR "behaviors"[All Fields] OR "pattern"[All Fields] OR "pattern s"[All Fields] OR "patternability"[All Fields] OR "patternable"[All Fields] OR "patterned"[All Fields] OR "patterning"[All Fields] OR "patternings"[All Fields] OR "patterns"[All Fields]) OR ("neurobehavior"[All Fields] OR "neurobehavioral"[All Fields] OR "neurobehaviors"[All Fields] OR "neurobehaviour"[All Fields] OR "neurobehavioural"[All Fields]) OR "emotional problems"[All Fields] OR ("mental"[All Fields] OR "mentalities"[All Fields] OR "mentality"[All Fields] OR "mentalization"[MeSH Terms] OR "mentalization"[All Fields] OR "mentalizing"[All Fields] OR "mentalize"[All Fields] OR "mentalized"[All Fields] OR "mentally"[All Fields]) OR ("temperament"[MeSH Terms] OR "temperament"[All Fields] OR "temperaments"[All Fields]) OR ("psychomotor"[All Fields] OR "psychomotoric"[All Fields]) OR ("motor"[All Fields] OR "motor s"[All	

	Fields] OR "motoric"[All Fields] OR "motorically"[All Fields] OR "motorics"[All Fields] OR "motoring"[All Fields] OR "motorisation"[All Fields] OR "motorised"[All Fields] OR "motorization"[All Fields] OR "motorized"[All Fields] OR "motors"[All Fields]) OR ("coordinate"[All Fields] OR "coordinated"[All Fields] OR "coordinately"[All Fields] OR "coordinates"[All Fields] OR "coordinating"[All Fields] OR "coordination"[All Fields] OR "coordinations"[All Fields] OR "coordinative"[All Fields] OR "coordinatively"[All Fields] OR "coordinator"[All Fields] OR "coordinator s"[All Fields] OR "coordinators"[All Fields]) OR "tic disorders"[All Fields] OR ("tourette syndrome"[MeSH Terms] OR ("tourette"[All Fields] AND "syndrome"[All Fields]) OR "tourette syndrome"[All Fields] OR "tourette"[All Fields] OR "tourettes"[All Fields] OR "tourette s"[All Fields] OR "tourettism"[All Fields]) OR ("communicate"[All Fields] OR "communicated"[All Fields] OR "communicates"[All Fields] OR "communicating"[All Fields] OR "communication"[MeSH Terms] OR "communication"[All Fields] OR "communications"[All Fields] OR "communicative"[All Fields] OR "communicational"[All Fields] OR "communicatively"[All Fields] OR "communicativeness"[All Fields] OR "communicator"[All Fields] OR "communicator s"[All Fields] OR "communicators"[All Fields]) OR ("language"[MeSH Terms] OR "language"[All Fields] OR "languages"[All Fields] OR "language s"[All Fields] OR "programming languages"[MeSH Terms] OR ("programming"[All Fields] AND "languages"[All Fields]) OR "programming languages"[All Fields]) OR ("speech"[MeSH Terms] OR "speech"[All Fields] OR "speeches"[All Fields]) OR ("anomia"[MeSH Terms] OR "anomia"[All Fields] OR "anomias"[All Fields]) OR ("dyslexia"[MeSH Terms] OR "dyslexia"[All Fields] OR "dyslexias"[All Fields]) OR ("learning"[MeSH Terms] OR "learning"[All Fields] OR "learn"[All Fields] OR "learned"[All Fields] OR "learning s"[All Fields] OR "learnings"[All Fields] OR "learns"[All Fields]) OR ("memories"[All Fields] OR "memory"[MeSH Terms] OR "memory"[All Fields] OR "memory s"[All Fields]) OR "executive function"[All Fields]	

3	"child*"[All Fields] OR "infant*"[All Fields] OR "school*"[All Fields] OR "postnatal"[All Fields] OR "postnatally"[All Fields] OR "prenatal"[All Fields] OR "prenatally"[All Fields] OR "prenatals"[All Fields] OR "post-natal"[All Fields] OR "pre-natal"[All Fields] OR "pregnan*"[All Fields] OR "mother s"[All Fields] OR "mothered"[All Fields] OR "mothers"[MeSH Terms] OR "mothers"[All Fields] OR "mother"[All Fields] OR "mothering"[All Fields]	10,123,559
4	#1 AND #2 AND #3	2,135
Scopus		
1	TITLE-ABS-KEY (arsenic OR arsenite OR arsenate)	141,380
2	TITLE-ABS-KEY (neurodevelopment* OR "developmental disorder" OR cognit* OR neurocognitive OR intell* OR academic OR "attention deficit hyperactivity disorder" OR adhd OR autism OR autistic OR autism AND spectrum AND disorder OR asperger OR behavior OR behaviour OR neurobehavioral OR "emotional problems" OR mental OR temperament OR psychomotor OR motor OR coordination OR "tic disorders" OR tourette OR communication OR language OR speech OR anomia OR dyslexia OR learning OR memory OR "executive function")	110,699
3	TITLE-ABS-KEY (child* OR infant* OR school* OR postnatal OR prenatal OR "post-natal" OR "pre-natal" OR pregnan* OR mother)	6,596,589
4	#1 AND #2 AND #3	71
Embase		
1	'arsenic'/exp OR arsenic OR 'arsenite'/exp OR arsenite OR 'arsenate'/exp OR arsenate	73,508
2	(neurodevelopment* OR 'developmental disorder' OR cognit* OR neurocognitive OR intell* OR academic OR 'attention deficit hyperactivity disorder' OR adhd OR autistic OR autism) AND spectrum AND disorder OR asperger OR behavior OR behaviour OR neurobehavioral OR 'emotional problems' OR mental OR temperament OR psychomotor OR motor OR coordination OR 'tic disorders' OR tourette OR communication OR language OR speech OR anomia OR dyslexia OR learning OR memory OR 'executive function'	6,006,070
3	child* OR infant* OR school* OR postnatal OR prenatal OR 'post-natal' OR 'pre-natal' OR pregnan* OR mother	12,833,451

4	#1 AND #2 AND #3	2,073
Web of Science		
1	arsenic OR arsenite OR arsenate (Abstract)	93,961
2	neurodevelopment* OR "developmental disorder" OR cognit* OR neurocognitive OR intell* OR academic OR "attention deficit hyperactivity disorder" OR ADHD OR autism OR autistic OR autism spectrum disorder OR Asperger OR behavior OR behaviour OR neurobehavioral OR "emotional problems" OR mental OR temperament OR psychomotor OR motor OR coordination OR "tic disorders" OR Tourette OR communication OR language OR speech OR anomia OR dyslexia OR learning OR memory OR "executive function" (Abstract)	15,951,012
3	child* OR infant* OR school* OR postnatal OR prenatal OR "post-natal" OR "pre-natal" OR pregnan* OR mother (Abstract)	4,462,304
4	#1 AND #2 AND #3	705
PsycINFO		
1	arsenic OR arsenite OR arsenate	326
2	neurodevelopment* OR "developmental disorder" OR cognit* OR neurocognitive OR intell* OR academic OR "attention deficit hyperactivity disorder" OR ADHD OR autism OR autistic OR autism spectrum disorder OR Asperger OR behavior OR behaviour OR neurobehavioral OR "emotional problems" OR mental OR temperament OR psychomotor OR motor OR coordination OR "tic disorders" OR Tourette OR communication OR language OR speech OR anomia OR dyslexia OR learning OR memory OR "executive function"	3,895,374
3	child* OR infant* OR school* OR postnatal OR prenatal OR "post-natal" OR "pre-natal" OR pregnan* OR mother	2,298,494
4	S1 AND S2 AND S3	106

Table S3: Characteristics of the most used assessment test for neuropsychological function.

Test name	Abbreviation	Age	Domain
Wechsler Intelligence Scale for Children	WISC	6-16 years	C
Bayley Scales of Infant Development	BSID	3-28 months	C, SE, P
Raven's Progressive Matrices	RPM	6-16 years	C
Behavior Assessment System for Children	BASC	6-11 years	SE
McCarthy Scale of Children's Ability	MSCA	2.5-8.5 years	C, P
Ages and Stages Questionnaire Inventory	ASQ	1 month - 5.5 years	C, SE, P
Bruininks–Oseretsky Test of Motor Proficiency	BOT	4-21 years	P
Child Behavior Checklist—Teacher's Report Form	CBCL	6-18 years	SE
Neonatal Behavioral Neurological Assessments	NBNA	birth-2 years	C, SE, P
Social Responsiveness Scale	SRS	2.5-18 years	SE
Woodcock–Munoz Cognitive Battery	WMCB	5-95 years	C
Behavior Rating Inventory of Executive Function	BRIEF	5-18 years	C
Behavioral Assessment and Research System	BARS	4-91 years	SE
Bender Visual–Motor Gestalt Test	BENDER	3-85 years	C, P
Rapid Visual Information Processing	RVP	> 6 years	C
Stockings of Cambridge	SOC	> 7 years	C
Intra-Dimensional/Extra-Dimensional Shift Task	IED	> 7 years	C
Spatial Span	SSP	> 6 years	C
Malawi Developmental Assessment Tool	MDAT	0-5 years	C, SE, P
Comprehensive Developmental Inventory for Infants and Toddlers	CDIIT	3-71 months	C, SE, P
Purdue Pegboard Test	PPT	> 5 years	P
Denver Developmental Screening Test II	DDST-II	birth-6 years	C, SE, P

C, cognition; SE, socio-emotional; P, psychomotor.

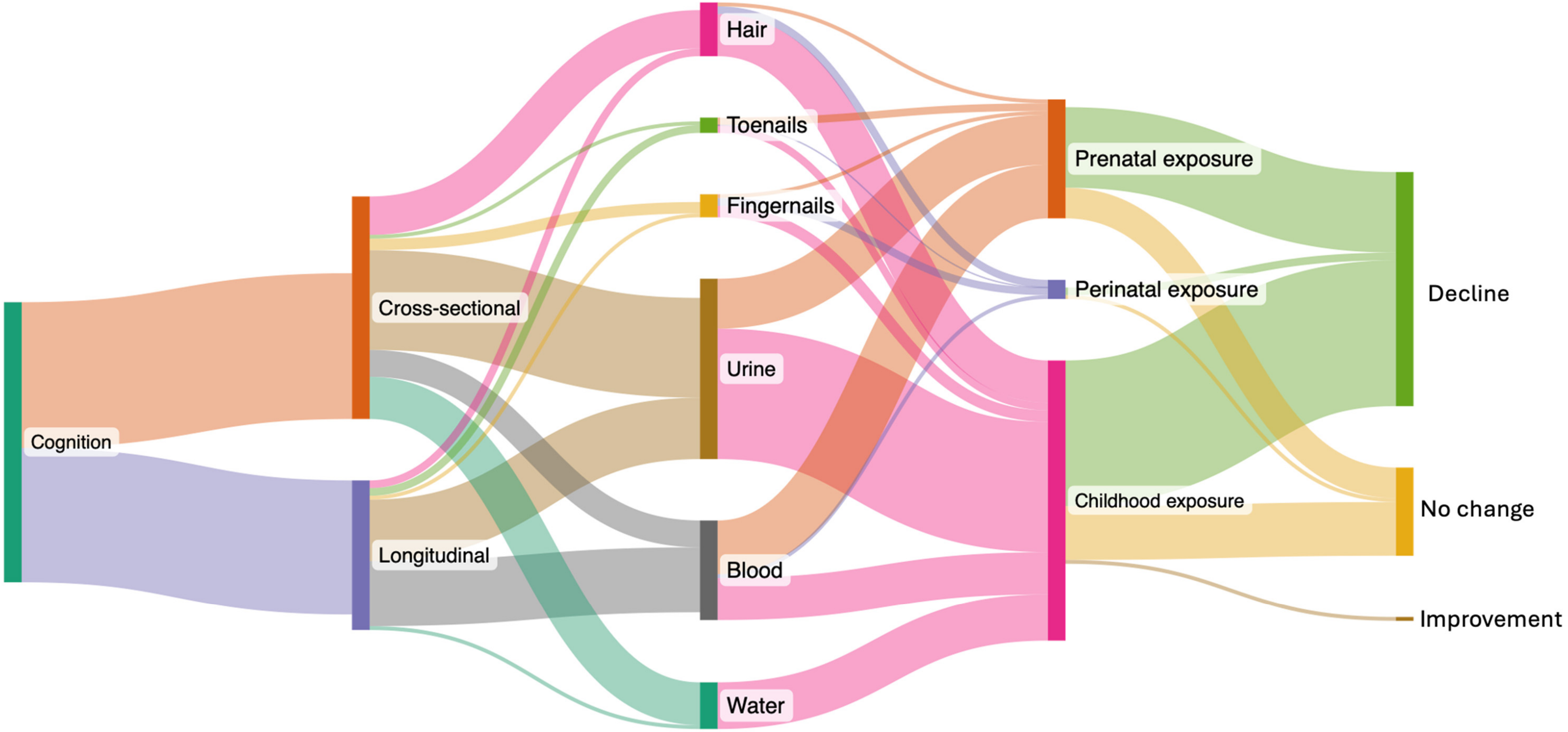

Figure S1: Sankey plot from the included studies assessing the cognition functional domain.

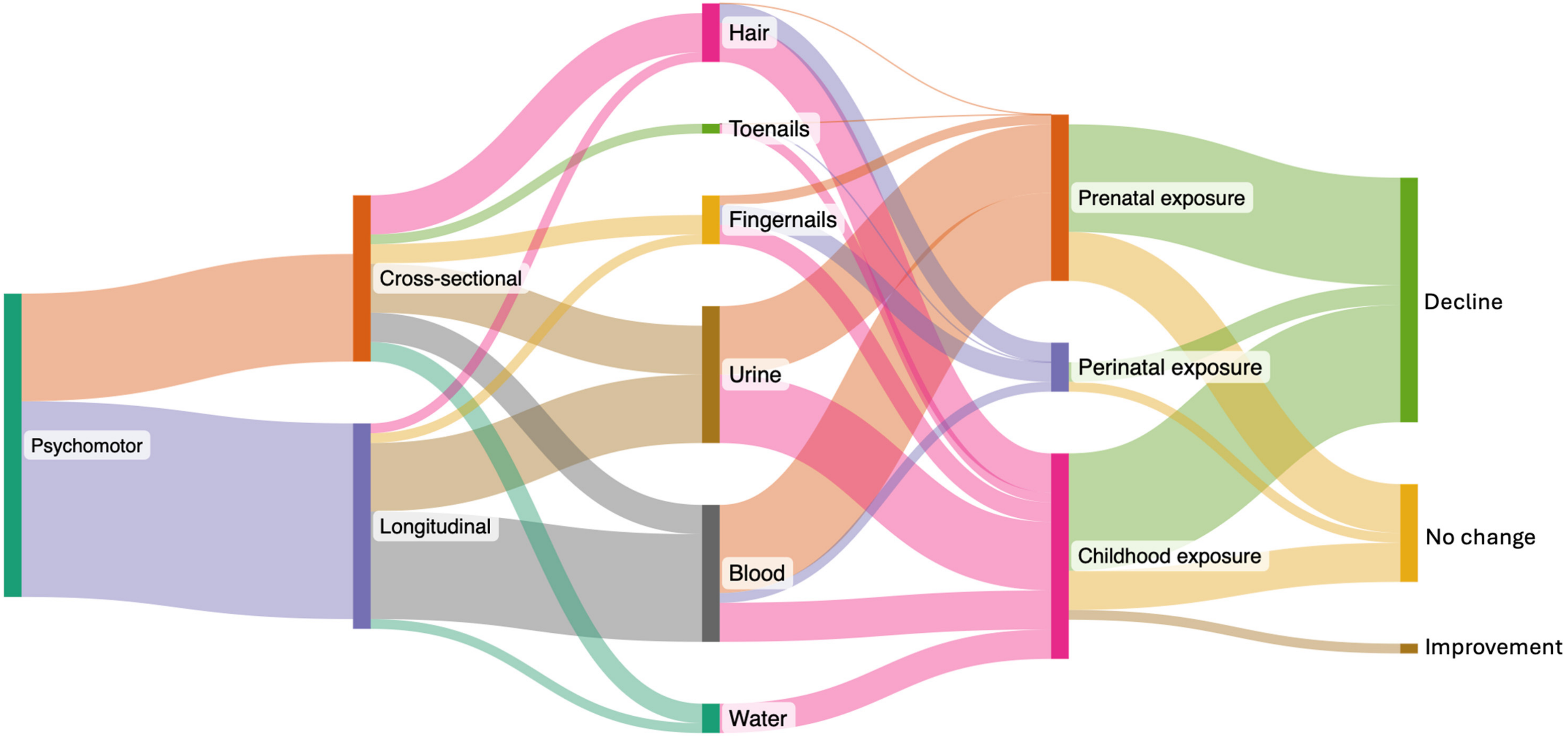

Figure S2: Sankey plot from the included studies assessing psychomotor functional domain.

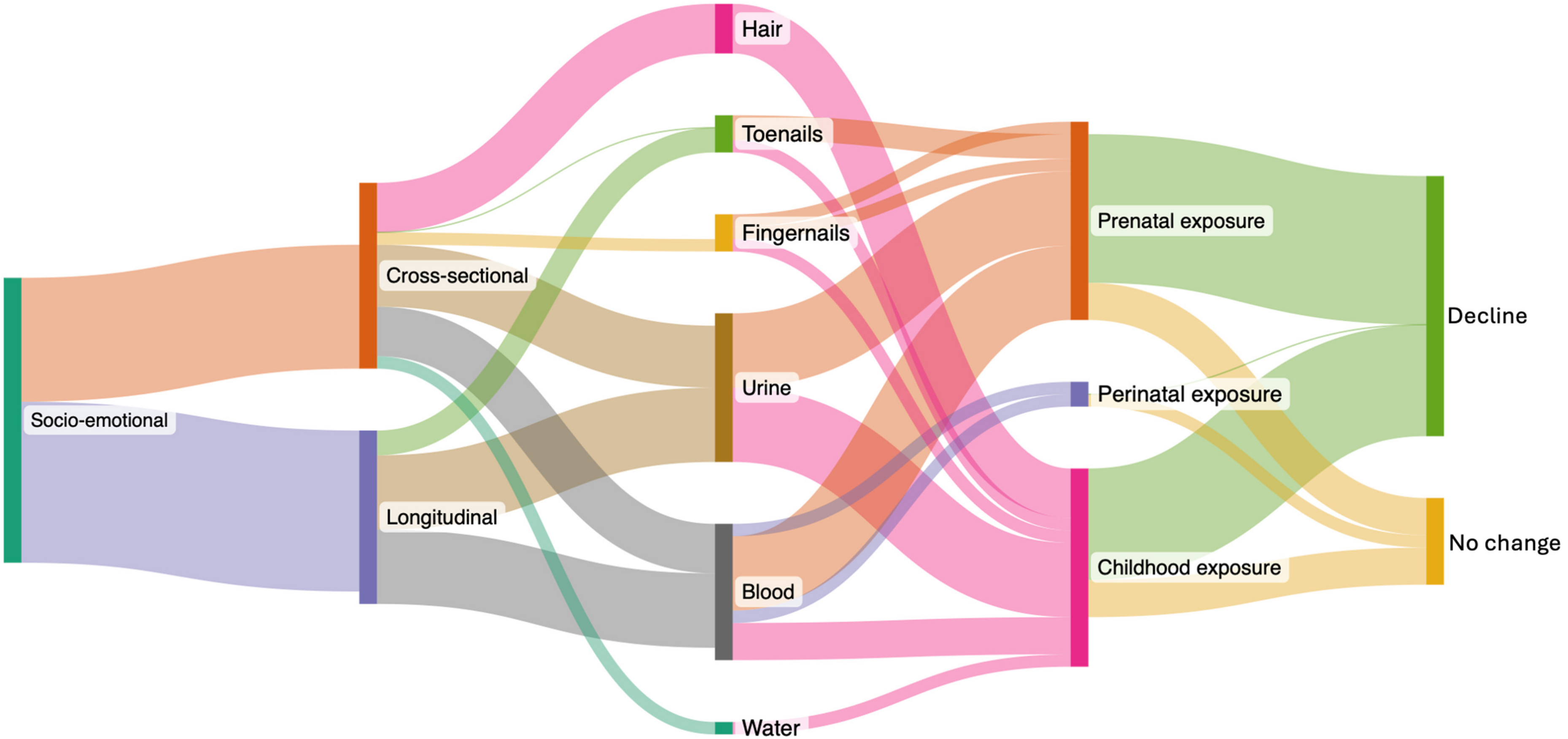

Figure S3: Sankey plot from the included studies assessing socio-emotional functional domain.
